# Comparative effects of hypoxic exercise training modalities on cardiometabolic health: A systematic review and network meta-analysis

**DOI:** 10.1016/j.isci.2025.114194

**Published:** 2025-11-22

**Authors:** Jin Huang, Bin Chen, Li Ding, Jue Liu, Li Guo, Yinhang Cao, Olivier Girard

**Affiliations:** 1 School of Athletic Performance, Shanghai University of Sport, Shanghai, China; 2 Department of Public Physical Education, Fujian Agriculture and Forestry University, Fuzhou, China; 3 Department of Rehabilitation Medicine, Huashan Hospital, Fudan University, Shanghai, China; 4 School of Exercise and Health, Shanghai University of Sport, Shanghai, China; 5 School of Human Sciences (Exercise and Sport Science), The University of Western Australia, Perth, Western Australia, Australia

**Keywords:** Health sciences, Medical science

## Abstract

While the benefits of various training modalities on cardiometabolic health and body composition are well-established under normoxic conditions, their efficacy in hypoxic environments remains unclear. We conducted a frequentist network meta-analysis (NMA) using random-effects models to evaluate the efficacy of hypoxic exercise modalities in individuals with excess body weight or obesity. Analysis revealed that combined hypoxic aerobic and resistance training is the most effective modality for improving body mass index, fat mass, and several cardiometabolic markers (e.g., low-density lipoprotein cholesterol, systolic blood pressure, and diastolic blood pressure). Hypoxic aerobic training is most beneficial for reducing triglycerides, while hypoxic high-intensity interval training is best for improving maximal oxygen consumption. This first NMA of hypoxic exercise establishes a hierarchy of modality-specific effects, enabling personalized prescription for individuals with excess body weight and obesity.

## Highlights

• Hypoxic-CT is the most effective for improving BMI, FM, LDL-C, SBP, and DBP
• Hypoxic-AT is most effective for reducing TG, while hypoxic-HIIT best improves VO_2max_

## Graphical abstract

**Figure:**
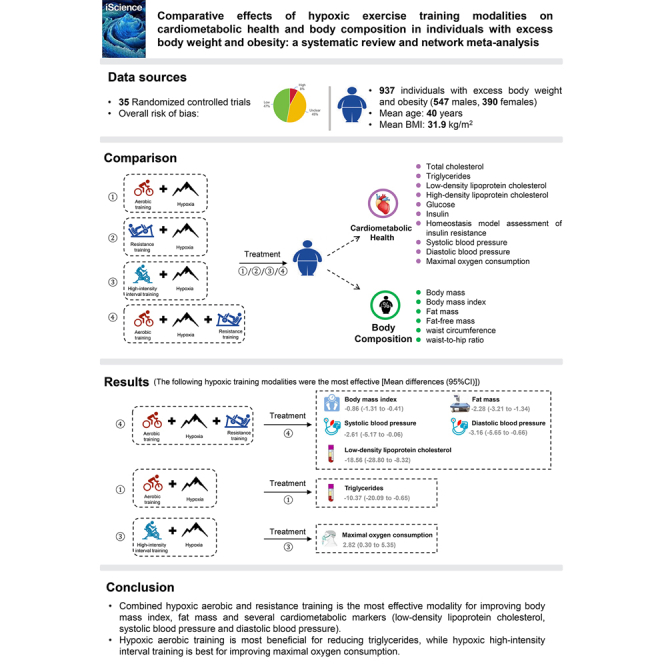

## Introduction

Obesity is a major global health concern linked to cardiometabolic disorders and type 2 diabetes.^1^^,^^2^ Regular exercise has been reported as a foundational component of obesity prevention, management, and treatment.^3^ For instance, aerobic training (AT),^4^ resistance training (RT),^5^ combined training (CT),^6^ and high-intensity interval training (HIIT)^7^ have been shown to improve body composition, cardiometabolic profiles, and physical fitness in individuals with or without metabolic disorders. Current guidelines recommend a minimum of 75–150 min of moderate-to-vigorous exercise weekly for optimal health.^8^^,^^9^ However, populations with excess body weight and adiposity may face an elevated risk of musculoskeletal injuries when exercising due to excess body weight.^10^^,^^11^ Thus, it is essential to identify safe, well-tolerated, and effective exercise regimens to improve cardiometabolic health and body composition in this population.

Hypoxia exposure reduces oxygen availability, meaning lower-intensity exercise under hypoxic conditions can induce comparable or even greater physiological stress than higher-intensity exercise in normoxia.^12^^,^^13^ This feature makes hypoxic exercise a promising strategy for populations with excess body weight and adiposity, as it provides effective training stimuli with lower mechanical load and reduced risk of joint injury.^14^^,^^15^^,^^16^ Consequently, hypoxic training has been explored as a potential intervention to improve body composition (e.g., body mass [BM], fat mass [FM], muscle mass [MM]) and cardiometabolic markers (e.g., triglycerides [TGs]) in individuals with excess body weight and obesity.^17^^,^^18^ Three recent meta-analyses of various hypoxic training modalities (e.g., AT, RT, CT, and HIIT) indicate trends toward improvements in BM (*p* = 0.07), TGs (*p* = 0.06–0.09), and MM (*p* = 0.08) in individuals with excess body weight and obesity compared with normoxia.^19^^,^^20^^,^^21^ However, these findings are likely influenced by considerable heterogeneity (*I*^2^ > 50%) across training modalities.^14^ For instance, hypoxic-CT and hypoxic-AT show greater reductions in BM,^18^^,^^22^^,^^23^ FM,^24^^,^^25^^,^^26^^,^^27^ and TGs^28^^,^^29^ than normoxic equivalents. Contrastingly, hypoxic-RT and hypoxic-HIIT do not show greater reductions in BM, FM, or TGs compared with normoxia.^17^^,^^30^^,^^31^ These findings suggest that the benefits of hypoxic training on cardiometabolic health and body composition may depend on the specific training modality used.^14^

Previous meta-analyses have mainly investigated single aerobic exercise modalities under hypoxic conditions in relation to body composition and metabolic health,^32^ leaving uncertainty about whether other exercise forms confer additional benefits. Moreover, traditional pairwise meta-analyses often aggregate different exercise types,^19^^,^^20^^,^^33^ which weakens the evidence and obscures differences in effectiveness among specific exercise modalities in hypoxic environments. To address these limitations, network meta-analysis (NMA) allows for a ranking of interventions by integrating both direct and indirect comparisons to derive more comprehensive and robust results.^34^^,^^35^ For instance, a recent NMA under normoxic conditions evaluated the effects of various training modalities on cardiometabolic markers and body composition, demonstrating a clear hierarchy in their effects on TGs (AT > HIIT > CT > RT) in individuals with excess body weight and obesity.^36^^,^^37^ However, no NMA has yet compared the effects of these modalities on health markers under hypoxic conditions.

Therefore, this NMA aimed to comprehensively evaluate and rank the effects of various hypoxic training modalities (e.g., AT, RT, CT, and HIIT) on cardiometabolic health and body composition in individuals with excess body weight and obesity.

## Results

### Studies included and characteristics

**Figure 1:**
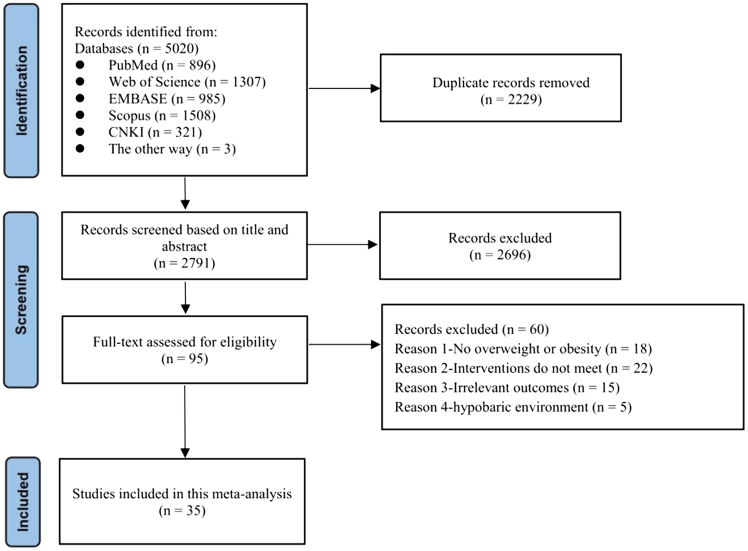
Flow diagram of study selection

The systematic review flow is presented in Figure 1. A total of 5,020 studies were identified from the databases. After removing 2,229 duplicates, 2,696 studies were excluded based on titles and abstracts. Sixty studies underwent full-text screening, and 35 studies met the inclusion criteria for analysis.

A total of 937 participants from 35 studies were included (474 in hypoxia, 463 in normoxia). The mean age was 40 ± 14 years, and the mean body mass index (BMI) was 31.9 ± 3.4 kg/m^2^. Thirteen studies (37%) included only males, six (17%) only females, while the remaining studies included both sexes. Two studies (5%) did not report sex. Fourteen studies (366 participants) examined hypoxic-AT, 11 (277 participants) investigated hypoxic-CT, 6 (176 participants) focused on hypoxic-HIIT, and 5 (108 participants) assessed hypoxic-RT. Exercise frequency ranged from 2 to 5 days per week, with durations from 2 to 32 weeks. In the hypoxia groups, inspired oxygen fraction (FiO_2_) ranged from 12.0% to 16.5%. A detailed summary of each study is presented in Supplement 2.

### Risk of bias

The percentage of studies with low, unclear, and high risk of bias for each item was as follows: random sequence generation (97.1%, 2.9%, and 0%, respectively), allocation concealment (8.6%, 91.4%, and 0%), blinding of participants and personnel (48.6%, 51.4%, and 0%), blinding of outcome assessors (5.7%, 94.3%, and 0%), incomplete outcome data (97.4%, 0%, and 2.9%), selective outcome reporting (100%, 0%, and 0%), and other risks of bias (48.6%, 45.7%, and 5.7%). Detailed risk of bias information is reported in Supplement 3.

### Network meta-analyses

**Figure 2:**
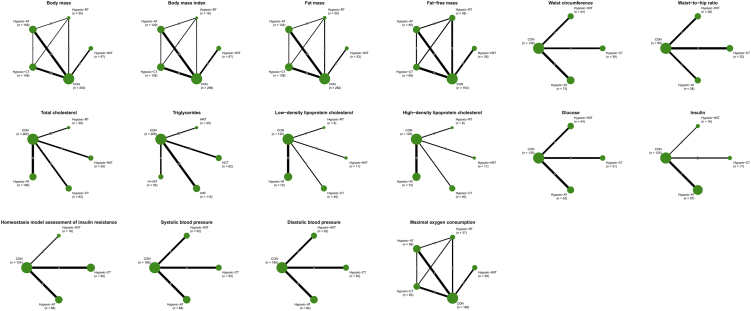
Network plots CON control; hypoxic-AT, hypoxic aerobic training; hypoxic-RT, hypoxic resistance training; hypoxic-CT, hypoxic resistance combined with hypoxic aerobic training; hypoxic-HIIT, hypoxic high-intensity interval training. The size of the nodes relates to the number of participants in that type of intervention, and the thickness of lines between interventions relates to the number of studies for that comparison.

**Figure 3:**
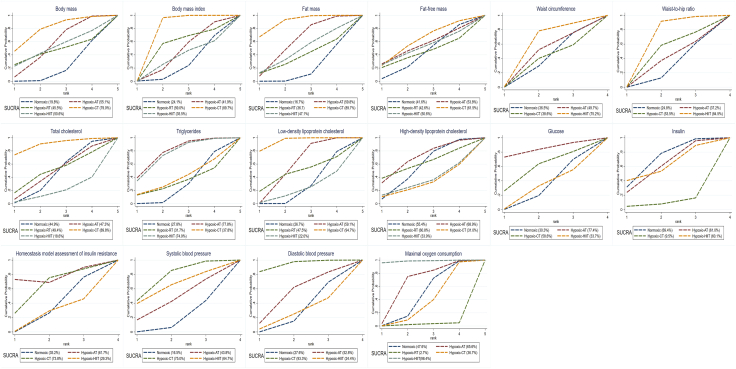
Cumulative probability plots CON, control; hypoxic-AT, hypoxic aerobic training; hypoxic-RT, hypoxic resistance training; hypoxic-CT, hypoxic resistance combined with hypoxic aerobic training; hypoxic-HIIT, hypoxic high-intensity interval training.

Network evidence plots for all indicators are presented in Figure 2. Network forest plots and league tables are provided in Supplement 4 and Table 1. Cumulative probability plots were created by combining the surface under the cumulative ranking probability curve values with line plots (Figure 3). The heterogeneity results are presented in Supplement 5. After reviewing baseline characteristics (sample size, proportion of males, mean age, and mean BMI), we confirmed the validity of the transitivity assumption (Supplement 6). There was no strong evidence of local inconsistencies in the network, with all *p* values exceeding 0.05 (Supplement 7). Comparison-adjusted funnel plots are shown in Supplement 8.

**Table 1:** League tables

	CON	−0.91 (−1.80, −0.02)	−0.58 (−5.96, 4.80)	−1.96 (−4.14, 0.21)	−0.92 (−4.57, 2.74)	BM
BMI	−0.13 (−0.45, 0.18)	Hypoxic-AT	0.33 (−5.09, 5.75)	−1.06 (−3.40, 1.29)	−0.01 (-3.77, 3.75)	
	−0.47 (−2.06, 1.13)	−0.33 (−1.95, 1.28)	Hypoxic-RT	−1.38 (−7.02, 4.26)	−0.34 (−6.84, 6.16)	
	**−0.86 (-1.31, -0.41)**	**−0.73 (-1.28, -0.18)**	−0.40 (−2.04, 1.25)	Hypoxic-CT	1.04 (−3.21, 5.30)	
	−0.06 (−1.19, 1.06)	0.07 (−1.10, 1.24)	0.40 (−1.55, 2.36)	0.80 (−0.41, 2.01)	Hypoxic-HIIT	
	CON	−1.30 (−2.62, 0.02)	−0.45 (-3.68, 2.79)	**−2.28 (-3.21, -1.34)**	−0.88 (−3.40, 1.63)	FM
FFM	0.25 (−2.31, 2.82)	Hypoxic-AT	0.85 (−2.51, 4.21)	−0.98 (−2.57, 0.61)	0.42 (−2.42, 3.26)	
	−0.09 (−3.25, 3.07)	−0.35 (−3.88, 3.19)	Hypoxic-RT	−1.83 (−5.15, 1.50)	−0.43 (−4.53, 3.66)	
	0.44 (−1.04, 1.91)	0.18 (−2.66, 3.02)	0.53 (−2.78, 3.84)	Hypoxic-CT	1.39 (−1.29, 4.08)	
	0.16 (−2.30, 2.62)	−0.09 (−3.64, 3.46)	0.25 (−3.75, 4.26)	−0.27 (−3.14, 2.59)	Hypoxic-HIIT	
	CON	−0.52 (−3.95, 2.91)	−0.03 (−4.38, 4.32)	−1.93 (−6.38, 2.51)		WC
WHR	0.00 (−0.05, 0.04)	Hypoxic-AT	0.49 (−5.05, 6.03)	−1.41 (−7.03, 4.21)		
	−0.01 (−0.06, 0.04)	−0.01 (−0.08, 0.06)	Hypoxic-CT	−1.90 (−8.12, 4.32)		
	−0.03 (−0.06, 0.00)	−0.03 (−0.08, 0.03)	−0.02 (−0.08, 0.04)	Hypoxic-HIIT		
	CON	−0.45 (−10.74, 9.84)	−0.95 (−19.55, 17.65)	−11.43 (−25.89, 3.02)	6.65 (−8.44, 21.73)	TC
TG	**−10.37 (-20.09, -0.65)**	Hypoxic-AT	−0.50 (−21.77, 20.76)	−10.99 (−28.49, 6.52)	7.09 (−11.14, 25.32)	
	2.60 (−24.29, 29.48)	12.97 (−15.62, 41.55)	Hypoxic-RT	−10.48 (−34.06, 13.09)	7.60 (−16.35, 31.55)	
	−0.28 (−23.59, 23.03)	10.09 (−15.16, 35.35)	−2.87 (−38.46, 32.71)	Hypoxic-CT	18.08 (−2.73, 38.89)	
	−9.82 (−20.51, 0.86)	0.55 (−13.90, 14.99)	−12.42 (−41.35, 16.51)	−9.55 (−35.19, 16.10)	Hypoxic-HIIT	
	CON	**−5.15 (-8.57, -1.72)**	−3.87 (−34.89, 27.15)	**−18.56 (-28.80, -8.32)**	3.87 (−15.04, 22.78)	LDL-C
HDL-C	−1.58 (−7.65, 4.48)	Hypoxic-AT	1.28 (−29.93, 32.48)	**−13.41 (-24.21, -2.62)**	9.02 (−10.21, 28.24)	
	−1.16 (−15.63, 13.31)	0.42 (−15.26, 16.11)	Hypoxic-RT	−14.69 (−47.35, 17.97)	7.74 (−28.59, 44.07)	
	4.25 (−8.58, 17.08)	5.83 (−8.36, 20.02)	5.41 (−13.92, 24.74)	Hypoxic-CT	22.43 (0.92, 43.94)	
	3.86 (−9.56, 17.28)	5.44 (−9.29, 20.17)	5.02 (−14.71, 24.75)	−0.39 (−18.96, 18.18)	Hypoxic-HIIT	
	CON	−4.02 (−10.42, 2.39)	−2.37 (−10.04, 5.30)	0.21 (−6.98, 7.40)		Glucose
Insulin	−1.07 (−4.79, 2.64)	Hypoxic-AT	1.65 (−8.28, 11.57)	4.23 (−5.70, 14.15)		
	5.00 (−3.92, 13.91)	6.07 (−3.60, 15.73)	Hypoxic-CT	2.58 (−8.13, 13.28)		
	0.30 (−8.82, 9.42)	1.37 (−8.48, 11.22)	−4.70 (−17.45, 8.06)	Hypoxic-HIIT		
	CON	−1.02 (−4.94, 2.91)	**−2.61 (-5.17, -0.06)**	−2.23 (−7.07, 2.61)		SBP
DBP	−0.98 (−3.98, 2.01)	Hypoxic-AT	−1.60 (−6.27, 3.08)	−1.22 (−7.44, 5.01)		
	**−3.16 (-5.65, -0.66)**	−2.17 (−6.05, 1.70)	Hypoxic-CT	0.38 (−5.09, 5.85)		
	0.31 (−3.15, 3.77)	1.29 (−3.29, 5.87)	3.46 (−0.82, 7.75)	Hypoxic-HIIT		
	CON	0.40 (−0.59, 1.40)	−2.56 (−5.39, 0.27)	−0.10 (−0.50, 0.31)	**2.82 (0.30, 5.35)**	VO_2max_
HOMA-IR	−0.15 (−0.53, 0.24)	Hypoxic-AT	−2.96 (−5.92, −0.00)	−0.50 (−1.57, 0.57)	2.42 (−0.29, 5.14)	
			Hypoxic-RT	2.46 (−0.38, 5.30)	**5.38 (1.59, 9.17)**	
	−0.32 (−1.12, 0.48)	−0.17 (−1.05, 0.71)		Hypoxic-CT	**2.92 (0.36, 5.48)**	
	0.10 (−0.60, 0.80)	0.25 (−0.55, 1.04)		0.42 (−0.64, 1.48)	Hypoxic-HIIT	

Effects are expressed as the effect size (95% CI) between interventions.

Bold indicates that the longitudinal intervention has a more significant reduction impact than the horizontal intervention or the horizontal intervention has a more significant impact than the longitudinal intervention.

CON, control; hypoxic-AT, hypoxic aerobic training; hypoxic-RT, hypoxic resistance training; hypoxic-CT, hypoxic resistance combined with hypoxic aerobic training; hypoxic-HIIT, hypoxic high-intensity interval training; WC, waist circumference; WHR, waist-to-hip ratio; TC, total cholesterol; HOMA-IR, homeostasis model assessment of insulin resistance.

### Cardiometabolic health

Total cholesterol, TGs, low-density lipoprotein cholesterol (LDL-C), high-density lipoprotein cholesterol (HDL-C), glucose, insulin, and homeostasis model assessment of insulin resistance were assessed in 20 (*n* = 525), 21 (*n* = 541), 9 (*n* = 266), 9 (*n* = 266), 11 (*n* = 303), 9 (*n* = 255), and 11 (*n* = 318) studies, respectively (Figure 2). NMA consistency analysis indicated that hypoxic-AT significantly improved TGs (mean difference [MD] = −10.37, 95% confidence interval [CI]: −20.09 to −0.65) and LDL-C (MD = −5.15, 95% CI: −8.57 to −1.72). Hypoxic-CT was more effective at reducing LDL-C than control (MD = −18.56, 95% CI: −28.80 to −8.32), hypoxic-AT (MD = −13.41, 95% CI: −24.21 to −2.62), and hypoxic-HIIT (MD = 22.43, 95% CI: 0.92 to 43.94) (Table 1). The Surface Under the Cumulative Ranking curve (SUCRA) probability ranking showed that hypoxic-AT had the highest probability of being the most effective intervention for TGs (SUCRA = 77.8%), while hypoxic-CT was best for LDL-C (SUCRA = 94.7%) (Figure 3). The quality of evidence for treatment rankings was low (Supplement 9). Pairwise meta-analysis results demonstrated that hypoxic-AT led to significant reductions in TGs (MD = −10.37, 95% CI: −20.09 to −0.65, *p* < 0.05, *I*^2^ = 0%) and LDL-C (MD = −5.15, 95% CI: −8.57 to −1.72, *p* < 0.01, *I*^2^ = 0%) compared to the same training under normoxic conditions. Hypoxic-CT resulted in significant reductions in LDL-C (MD = −18.56, 95% CI: −28.80 to 8.32, *p* < 0.01) compared to normoxic training (Supplement 9).

Systolic blood pressure (SBP), diastolic blood pressure (DBP), and maximal oxygen consumption (VO_2max_) were assessed in 15 (*n* = 373), 15 (*n* = 373), and 18 studies (*n* = 415) studies, respectively (Figure 2). NMA consistency analysis indicated that hypoxic-CT improved SBP (MD = −2.61, 95% CI: −5.17 to −0.06) and DBP (MD = −3.16, 95% CI: −5.65 to −0.66) compared to control. Hypoxic-HIIT was significantly more effective at improving VO_2max_ than control (MD = 2.82, 95% CI: 0.30 to 5.35), hypoxic-RT (MD = 5.38, 95% CI: 1.59 to 9.17), and hypoxic-CT (MD = 2.92, 95% CI: 0.36 to 5.48) (Table 1). The SUCRA probability ranking showed that hypoxic-CT had the highest probability of being the most effective intervention for SBP (SUCRA = 75.0%), and DBP (SUCRA = 93.3%), while hypoxic-HIIT was most effective for VO_2max_ (SUCRA = 98.4%) (Figure 3). The quality of the evidence for these treatment rankings was low (Supplement 9). Pairwise meta-analysis showed that hypoxic-CT led to significant reductions in SBP (MD = −2.61, 95% CI: −5.17 to −0.06, *p* < 0.05, *I*^2^ = 0%) and DBP (MD = −3.09, 95% CI: −5.63 to −0.55, *p* < 0.05, *I*^2^ = 15%) compared to the same training under normoxic conditions. Hypoxic-HIIT resulted in significant improvement in VO_2max_ (MD = 2.82, 95% CI: 0.30 to 5.35, *p* < 0.05, *I*^2^ = 0%) compared to normoxic training (Supplement 9).

### Body composition

BM, BMI, FM, fat-free mass (FFM), waist circumference and waist-to-hip ratio were assessed in 33 (*n* = 747), 28 (*n* = 639), 24 (*n* = 488), 19 (*n* = 453), 11 (*n* = 304), and 7 (*n* = 178) studies, respectively (Figure 2). NMA consistency analysis indicated that hypoxic-AT (MD = −0.91, 95% CI: −1.80 to −0.02) led to significant reduction in BM compared to control. Hypoxic-CT (MD = −0.86, 95% CI: −1.31 to −0.41) was significantly more effective at reducing BMI than both control and hypoxic-AT (MD = −0.73, 95% CI: −1.28 to −0.18). Hypoxic-CT (MD = −2.28, 95% CI: −3.21 to −1.34) was significantly more effective than control at reducing FM (Table 1). The SUCRA probability ranking showed that hypoxic-CT had the highest probability of being the most effective intervention for BMI (SUCRA = 89.7%) and FM (SUCRA = 89.7%) (Figure 3). The quality of evidence for treatment rankings was low (Supplement 9). Pairwise meta-analysis results demonstrated that hypoxic-CT resulted in significant reductions in BMI (MD = −0.88, 95% CI: −1.33 to −0.42, *p* < 0.01, *I*^2^ = 0%) and FM (MD = −2.32, 95% CI: −3.27 to −1.37, *p* < 0.01, *I*^2^ = 0%) than the same training under normoxic conditions (Supplement 9).

### Meta-regression

Meta-regression results are presented in Supplement 10. When comparing hypoxic-AT and hypoxic-CT with control for outcomes including BM, BMI, FM, and TGs, no significant moderating effects were observed for sample size, year of publication, proportion of females, age, BMI, hypoxia severity, hypoxia duration, exercise frequency, and training cycles.

## Discussion

This study is the first to use NMA to comprehensively evaluate and rank the effects of various hypoxic training modalities (AT, RT, CT, and HIIT) on cardiometabolic health and body composition in individuals with excess body weight and obesity. The findings indicate that hypoxic-CT emerged as the most effective regimen for improving BMI, FM, LDL-C, SBP, and DBP. Additionally, hypoxic-AT and hypoxic-HIIT were the most beneficial for reducing TG and boosting VO_2max_, respectively. Finally, hypoxic training across AT, CT, and HIIT produced greater improvements in cardiometabolic health (TGs, LDL-C, SBP, DBP, and VO_2max_) and body composition (BMI and FM) at lower exercise intensities compared to training in normoxia.

### Cardiometabolic health outcomes

Our network analytical approach revealed that hypoxia-AT had the highest probability of being the most effective intervention for improving blood lipid profiles (TGs and LDL-C) (Figure 3; Table 1). This aligns with previous NMA performed under normoxic conditions.^36^ Notably, the exercise intensity of hypoxic-AT in our study was generally lower than prior NMA (60%–75% vs. 70%–85% HR_max_) (Supplement 2).^36^ A traditional meta-analysis further confirmed that lower-intensity hypoxic-AT led to greater improvements in TG (MD = −10.37 mg/dL, *p* < 0.05) and LDL-C (MD = −5.15 mg/dL, *p* < 0.05) levels compared to normoxic AT (88–96 W vs. 104–118 W) (Supplement 9). These results suggest that AT is the most effective intervention for improving lipid profiles in both normoxic and hypoxic conditions, with lower-intensity hypoxic AT resulting in superior benefits. AT is known to induce skeletal muscle adaptations (i.e., mitochondrial biogenesis and enhanced oxidative capacity), which improves fatty acid transport into mitochondria for oxidation.^38^^,^^39^^,^^40^ Hypoxia may further enhance muscle fatty acid uptake and oxidation, alongside increased lipoprotein lipase activity, which aids TG breakdown and improves blood lipid profiles during AT.^41^^,^^42^ The combination of hypoxic exposure and AT thus synergistically improves aerobic capacity and blood lipid profiles (TG and LDL-C). In addition to aerobic exercise, our traditional meta-analysis showed that HIIT and CT under hypoxic conditions led to greater improvements in lipid metabolism than in normoxia (MD_TG_ = −9.82 mg/dL, *p* = 0.07; MD_LDL-C_ = −18.56 mg/dL, *p* < 0.05) (Supplement 9). These findings support combining exercise with hypoxia as an effective strategy for obesity management. Individuals with obesity are at a higher risk of dyslipidemia.^43^ Evidence indicates that high-intensity aerobic exercise (>60% $\dot{V}$O_2max_ or >60% HRR) improves lipid metabolism (e.g., LDL-C, HDL-C), whereas moderate- and low-intensity exercise produce limited effects.^44^^,^^45^ Notably, high-intensity exercise often carries a higher risk of musculoskeletal injury in individuals with excess body weight and adiposity.^46^ This study proposes hypoxic conditioning as a safer and effective strategy to enhance lipid metabolism at relatively lower exercise intensities in this population.

SBP and DBP are well-established indicators of cardiovascular health and are associated with increased incidence and mortality from cardiovascular diseases.^47^^,^^48^ Consistent with previous NMA performed under normoxic conditions,^36^ our results showed that hypoxic-CT is the most effective intervention for reducing SBP and DBP (Figure 3; Table 1). Similar to hypoxic-AT, hypoxic-CT achieved greater blood pressure reductions (MD_SBP_ = −2.61 mmHg, *p* = 0.05; MD_DBP_ = −3.09 mmHg, *p* < 0.05) at lower exercise intensities (156–208 W vs. 184–238 W) compared to normoxic conditions (Supplement 9). The aerobic component of CT enhances vascular and endothelial function through vasodilation,^49^^,^^50^ while the resistance component enhances endothelial function by mobilizing endothelial progenitor cells.^50^ Hypoxia further amplifies these effects by increasing nitric oxide release, reducing sympathetic nervous system activity,^25^^,^^51^ and enhancing muscle metabolic activity and vasodilation during both AT and RT.^52^^,^^53^^,^^54^ Therefore, the combination of hypoxia and CT may lead to a stronger synergistic effect on blood pressure reduction. Our traditional meta-analyses indicated that AT (MD_SBP_ = −11.02 mmHg, *p* = 0.61; MD_DBP_ = −1.00 mmHg, *p* = 0.50) and HIIT (MD_SBP_ = −2.18 mmHg, *p* = 0.39; MD_DBP_ = 0.32 mmHg, *p* = 0.85) under hypoxic conditions produced only limited improvements compared with normoxia (Supplement 9). Evidence suggests these effects on blood pressure regulation are less pronounced than those observed with CT.^36^ This variability in blood pressure responses across exercise modalities may reflect differences in physiological adaptations (e.g., endothelial function and autonomic regulation).^55^

Growing evidence indicates that enhanced cardiorespiratory fitness, reflected by VO_2max_,^56^ can significantly mitigate the adverse effects of adiposity and other cardiovascular risk factors.^57^ Our study examined the effect of hypoxic training on VO_2max_ in individuals with excess body weight and obesity. In line with previous NMA conducted under normoxic conditions,^58^ hypoxic-HIIT emerged as the most effective intervention for improving VO_2max_ (Figure 3; Table 1). Like hypoxic-AT and hypoxic-CT, hypoxic-HIIT achieved these improvements (MD = 2.80 mL/kg/min, *p* = 0.03) at lower exercise intensities compared to normoxic HIIT (96–176 vs. 145–221 W) (Supplement 9). HIIT increases capillary and mitochondrial density, enhancing skeletal muscle oxygen uptake and/or muscle diffusing capacity.^59^^,^^60^ Under hypoxic conditions, these adaptations may be amplified via activation of hypoxia-inducible factors, promoting mitochondrial biogenesis and capillary growth,^61^^,^^62^ thus leading to greater VO_2max_ gains. Our meta-analysis also showed that AT under hypoxic conditions improved VO_2max_ (MD = 0.37 mL/kg/min, *p* = 0.47), although to a smaller extent than HIIT (0.37 vs. 2.80 mL/kg/min) (Supplement 9). This aligns with previous evidence that HIIT tends to produce greater increases in VO_2max_ compared with moderate-intensity AT.^63^^,^^64^

### Body composition outcomes

Under normoxic conditions, previous studies have demonstrated that CT improves cardiometabolic health in individuals with excess body weight or adiposity (e.g., enhancing lipid metabolism and regulating blood glucose),^65^^,^^66^^,^^67^ while also enhancing body composition (e.g., reducing body fat percentage).^68^^,^^69^ Our findings showed that hypoxic-CT achieved greater reductions in FM (MD = −2.32 kg, *p* < 0.01) and BMI (MD = −0.88 kg/m^2^, *p* < 0.01) at lower exercise intensities (156–208 vs. 184–238 W) compared to normoxia. Moreover, hypoxic-CT emerged as the most effective intervention for reducing FM and BMI (Figure 3; Table 1), which is consistent with earlier NMA under normoxic conditions.^36^^,^^58^ These effects may reflect the activation of several energy metabolism pathways. The aerobic component of CT primarily promotes fat oxidation by enhancing cardiorespiratory fitness and aerobic capacity,^56^^,^^70^ while the resistance component targets muscle hypertrophy and increases resting metabolic rate.^71^^,^^72^ Hypoxia may further amplify these effects by enhancing fat oxidation and energy expenditure during AT^73^^,^^74^^,^^75^ and stimulating growth hormone secretion during RT.^76^^,^^77^ This integrated approach at lower exercise intensities likely enhances the afterburn effect, leading to reductions in FM and BMI, while preserving FFM. Our traditional meta-analysis also demonstrated that AT under hypoxic conditions reduced FM (MD = −1.22 kg, *p* = 0.08) more than under normoxia. Therefore, for individuals with obesity seeking fat loss, hypoxic AT or CT represents an effective strategy.

### Conclusion

This NMA reveals that among hypoxic training modalities (AT, RT, CT, and HIIT), hypoxic-CT is most effective for improving BMI, FM, LDL-C, SBP, and DBP, while hypoxic-AT and hypoxic-HIIT are most beneficial for reducing TGs and improving VO_2max_, respectively. Furthermore, AT, CT, and HIIT under hypoxic conditions lead to greater improvements in cardiometabolic health (TGs, LDL-C, SBP, DBP, and VO_2max_) and body composition (BMI and FM) at lower exercise intensities compared to their normoxic counterparts. These findings offer practical guidance for clinicians in tailoring hypoxic exercise prescriptions for individuals with excess body weight and obesity. Further research is warranted to confirm whether this hierarchy is applicable across diverse populations.

### Limitations of the study

This study has several limitations. First, some comparisons were based on a small number of studies, with certain interventions (e.g., hypoxic-CT for LDL-C and hypoxic-HIIT for VO_2max_) represented by only one to three studies. Therefore, these findings should be interpreted with caution.^78^ Second, while the superiority of hypoxic-CT was demonstrated, the potential impact of the sequence of AT and RT was not considered. Prior research suggests that performing AT before RT may increase total energy expenditure and enhance fat loss through improved metabolic efficiency.^79^ Third, meta-regression analysis was limited to outcomes (i.e., BM, BMI, FM, and TGs) with data from at least 10 studies. The uneven distribution limited our ability to assess the influence of potential moderators—such as sample size, year of publication, proportion of females, age, BMI, hypoxia severity, hypoxia duration, exercise frequency, and training cycles—on other cardiometabolic health and body composition outcomes.^80^

## Resource availability

### Lead contact

Further information and requests for resources can be directed to and would be fulfilled by the lead contact, Yinhang Cao (caoyinhang@sus.edu.cn).

### Materials availability

This study did not generate new unique reagents.

### Data and code availability

• This study is a systematic review and network meta-analysis. The data analyzed in this study were extracted from previously published studies and publicly available resources. Raw data used for meta-analysis are available upon request.
• The meta-analysis was conducted in R using the standard function available in the *netmeta* package, and no custom code was developed. As a result, code sharing is not necessary for reproducing our results.
• Any additional information required to reanalyze the data reported in this paper is available from the lead contact upon request.

## Acknowledgments

We sincerely appreciate the participants for their valuable time and diligent effort in this study. This study was supported by the Shanghai Key Laboratory of Human Performance at 10.13039/501100002397Shanghai University of Sport (grant number: 11DZ2261100) and Sports and Fitness Technology Provincial and Ministerial Co-constructed Key Laboratory of the Ministry of Education Open Fund of 10.13039/501100002397Shanghai University of Sport (grant number: 2025KF0005).

## Author contributions

J.H. and L.D. conceived and designed the study. J.H. contributed to data collection. J.H. and B.C. performed data analysis. J.H., J.L., L.D., L.G., and Y.C. interpreted the experimental results. J.H. prepared the figures. J.H. and L.D. drafted the manuscript. J.H., L.D., B.C., L.G., O.G., and Y.C. edited and revised the manuscript. All authors read and approved the final manuscript.

## Declaration of interests

We declare no competing interests.

## STAR★Methods

### Key resources table

**Table:** 

REAGENT or RESOURCE	SOURCE	IDENTIFIER
**Deposited data**		

Studies For Meta-analysis	EMBASE	https://www.embase.com/
Studies For Meta-analysis	PubMed	https://pubmed.ncbi.nlm.nih.gov/
Studies For Meta-analysis	Web of Science	https://www.webofscience.com/wos/author/search
Studies For Meta-analysis	CNKI	https://www.cnki.net/
Studies For Meta-analysis	Scopus	https://www.scopus.com/
International prospective register of systematic reviews	PROSPERO	https://www.crd.york.ac.uk/PROSPERO/

**Software and algorithms**		

Stata software Version 16.0	Downloaded STATA software	https://www.stata.com/products
EndNote X9	Clarivate Analytics	https://endnote.com/downloads
R Version 4.1.1	Downloaded R	https://www.r-project.org/

### Experimental model and study participant details

Our study does not use experimental models typical in the life sciences.

### Method details

This NMA was conducted following the Preferred Reporting Items for Systematic Reviews and Meta-Analyses for Network Meta-Analyses (PRISMA-NMA),^81^ and registered with the International Prospective Register of Systematic Reviews (PROSPERO) database (CRD42024609489).

#### Search strategy

Five databases (PubMed, Web of Science, EMBASE, Scopus, and CNKI) were searched from inception to March 2025 using subject headings, keywords, and medical subject headings (MeSH) term searches for “hypoxia”, “exercise”, “body composition”, “cardiometabolic”, “excess body weight”, and “obesity” (detailed search strategy is presented in Supplement 1). When necessary, authors were contacted via email for missing data. Manual searches were conducted using Google Scholar to ensure comprehensive coverage of relevant literature.

#### Eligibility criteria

Each study had to meet participants, interventions, comparators, outcomes, and study design (PICOS) criteria: (1) participants classified as having excess body weight or obesity, defined as body mass index [BMI] > 25 kg/m^2^ (excess body weight) and/or ≥ 30 kg/m^2^ (excess adiposity) for Europeans, and BMI ≥ 24 kg/m^2^ (excessive weight) and/or BMI ≥ 28 kg/m^2^ (excess adiposity) for Asians. If BMI was not provided, body fat percentage was used as a criterion: ≥ 30 for females and ≥ 25 for males^56^; (2) the intervention involved aerobic training, resistance training, combined aerobic and resistance training, or high-intensity interval training in hypoxia (simulated altitude ≥ 2000 m or FiO_2_ < 16.4%)^82^); (3) All training modalities conducted under normoxic conditions were pooled as a common comparator^83^^,^^84^; (4) outcomes included measures of body composition (BM, BMI, FM, fat-free mass [FFM], waist circumference [WC], waist-to-hip ratio [WHR]) and cardiometabolic health (total cholesterol [TC], TG, low-density lipoprotein cholesterol [LDL-C], high-density lipoprotein cholesterol [HDL-C], glucose, insulin, homeostatic model assessment for insulin resistance [HOMA-IR], systolic blood pressure [SBP], diastolic blood pressure [DBP], maximal oxygen consumption [VO_2max_]) were reported; (5) study design was limited randomized controlled trials.

Studies were excluded based on the following criteria: (1) participants had physical activity restrictions or conditions that could impede exercise interventions; (2) the intervention period was less than two weeks^85^; (3) blood flow restriction was used as the hypoxic intervention; (4) full-text or data extraction was unavailable; (5) the study was a conference abstract, review, case report, or animal experiment; (6) the study was not published in English or Chinese.

#### Study selection and data extraction

Two investigators (HJ and ZC) independently screened titles and abstracts to identify potentially eligible studies, followed by full-text review. A third investigator (CY) resolved any disagreements regarding study inclusion.

Data from the included studies were independently extracted by pairs of investigators, focusing on: (1) basic information (author, year of publication, country); (2) participant characteristics (sample size, sex, age, and BMI); (3) hypoxic exposure (hypoxia severity, duration, and pattern); (4) protocol and training characteristics (training mode, volume, intensity, frequency, duration [number of weeks], and rest); (5) outcome indicators (mean, standard deviation (SD), and sample size) for each group before and after the intervention. Mean differences (MD) were calculated by subtracting pre-intervention means from post-intervention means for each group. The SD of the difference in means was computed using the following formula (assuming a correlation coefficient of 0.50)^86^:

$$\text{SDdiff}=\sqrt{\;{SD}_{pre}^{2}+{SD}_{post}^{2}-2r\times {SD}_{pre}\times {SD}_{post}}$$

where SD_diff_ is the standard deviation of the pre- and post-intervention differences, SD_pre_ and SD_post_ are the standard deviations for pre- and post-intervention measures, and *r* is the correlation coefficient between pre and post-intervention measurements.

#### Risk of bias assessment and GRADE

The risk of bias for each study was independently assessed by HJ and DL using the Cochrane Collaboration Risk of Bias Tool,^87^ This tool evaluates potential biases in the following areas: selection bias (random sequence generation and allocation concealment), performance bias (blinding of patients and personnel), detection bias (blinding of outcome assessment), attrition bias (incomplete outcome data), reporting bias (selective outcome reporting), and other bias. Each domain was rated as ‘low risk’, high risk’, or ‘unclear risk’. Any disagreements regarding the risk of bias were resolved by CY. The Grading of Recommendations Assessment, Development and Evaluation (GRADE) approach was used to assess the quality of evidence supporting the ranking of treatments from NMA.^88^

### Quantification and statistical analysis

Data analysis was performed using Stata software (Version 16.0; StataCorp, College Station, TX, USA) and R software (version 4.1.1; The R Foundation for Statistical Computing). All outcomes were converted to standard units, with continuous variables reported as mean (SD).^36^^,^^56^ Network meta-analysis (NMA) for all outcomes was conducted based on the frequentist framework using Stata software.^89^^,^^90^ A random-effects NMA was performed to calculate effect estimates, with mean difference (MD) and 95% confidence interval (CI) reported. Network evidence diagrams were constructed, with nodes representing interventions, line thickness proportional to the number of studies, and node size proportional to sample size. Forest plots and league tables of relative effects were used to visualize comparisons of network estimations. Cumulative probability plots illustrated the cumulative ranking of intervention effects, with higher surface-under-the-curve values indicating better effects.^91^ Global and local statistical heterogeneity were assessed with generalised Cochran’s Q.^92^ Local inconsistency of direct and indirect results was assessed with the side-splitting method.^93^ Transitivity assumptions were checked by comparing distributions of characteristics across study arms grouped by training modalities. Random-effects meta-regression analyses were conducted based on sample size, year of publication, proportion of females, age, BMI, hypoxia severity, hypoxia duration, exercise frequency, and training cycles. Publication bias was assessed using comparison-adjusted funnel plots and Egger’s test.^94^

Pairwise random-effects meta-analysis was conducted to compare the effects of the same exercise under hypoxic and normoxic conditions. Heterogeneity was assessed for all pairwise comparisons using the *I*^2^ statistic, and publication bias was evaluated with Egger’s test *p*-value.
