# Supplementary material for: Comparative effects of hypoxic exercise training modalities on cardiometabolic health: A systematic review and network meta-analysis
Source: iScience. 2025 Nov 22;28(12):114194. doi: 10.1016/j.isci.2025.114194 (PMC12722976; doi:10.1016/j.isci.2025.114194)
Supplement: Document S1. Supplements 1–10 [file mmc1.pdf]

iScience, Volume *28*

Supplemental information

Comparative effects of hypoxic exercise training modalities on cardiometabolic health: A systematic review and network meta-analysis

Jin Huang, Bin Chen, Li Ding, Jue Liu, Li Guo, Yinhang Cao, and Olivier Girard

Supplemental files

Contents

Supplement 1: Search strategy 2
Supplement 2: Study characteristics 3
Supplement 3: Risk of bias 10
Supplement 4: Forest plots 11
Supplement 5: Heterogeneity assessments 13
Supplement 6: Intransitivity assessments 21
Supplement 7: Inconsistency analysis 22
Supplement 8: Comparison-adjusted funnel plots 24
Supplement 9: Summary of pairwise meta-analyses 26
Supplement 10: Meta regression 28

Supplement 1: Search strategy

Step	**Search Strategy**
#1	"Hypoxia"[MeSH Terms] OR "hypoxi*"[Title/Abstract] OR "normoxi*"[Title/Abstract] OR "intermittent hypoxia"[Title/Abstract] OR "Altitude"[Title/Abstract] OR "oxygen deficiency"[Title/Abstract] OR "low oxygen levels"[Title/Abstract] OR "oxygen deprivation"[Title/Abstract] OR "live low train high"[Title/Abstract]
#2	"Obesity"[MeSH Terms] OR "Obesity"[Title/Abstract] OR "overweight"[Title/Abstract] OR "weight loss"[Title/Abstract] OR "fat loss"[Title/Abstract] OR "corpulence"[Title/Abstract] OR "adiposity"[Title/Abstract]
#3	"Body composition"[MeSH Terms] OR "body mass"[Title/Abstract] OR "body weight"[Title/Abstract] OR "body mass index"[Title/Abstract] OR "lean body mass"[Title/Abstract] OR "waist circumference"[Title/Abstract] OR "hip circumference"[Title/Abstract] OR "waist to hip ratio"[Title/Abstract] OR "fat mass"[Title/Abstract]
#4	"Metabolism"[MeSH Terms] OR "total cholesterol"[Title/Abstract] OR "triglyceride"[Title/Abstract] OR "low density lipoprotein cholesterol"[Title/Abstract] OR "high density lipoprotein cholesterol"[Title/Abstract] OR "low density lipoprotein"[Title/Abstract] OR "high density lipoprotein"[Title/Abstract] OR "Glucose"[Title/Abstract] OR "Insulin"[Title/Abstract] OR "cardiometabolic"[Title/Abstract] OR "cardiovascular risk"[Title/Abstract]
#5	#3 OR #4
#6	#1 AND #2 AND #5

Supplement 2: Study characteristics

Study	Country	Characteristics of participants: Sample size (M/F)	Age (years)	BMI	Interventions details: Hypoxic training protocol	Period and frequency (week × [times/week])	Type and severity of hypoxia	Pattern and duration of hypoxic exposure	Outcomes
Netzer et al., 2008	Germany	H: 10 (2/8)<br>N: 10 (2/8)	H: 50.1 ± 13.0<br>N: 45.5 ± 13.3	H: 33.4 ± 3.1<br>N: 32.8 ± 3.3	Hypoxic-AT, 90 min at 60% of the HR_{max} stepper, treadmill, bicycle ergometer	8 × 3	Normobaric,<br>FiO_2 = 15.0%	Intermittent,<br>90 min/session	①⑧
Haufe et al., 2008	Germany	H: 10 (10/0)<br>N: 10 (10/0)	H: 29.0 ± 5.9<br>N: 28.1 ± 5.2	H: 25.1 ± 1.9<br>N: 24.0 ± 1.6	Hypoxic-AT, 60 min of running at 3 mmol/L lactate	4 × 3	Normobaric,<br>FiO_2 = 15.0%	Intermittent,<br>60 min/session	①②⑧⑬⑯
Wiesner et al., 2010	Germany	H: 24 (10/14)<br>N: 21 (8/13)	H: 42.2 ± 1.2<br>N: 42.1 ± 1.7	H: 33.1 ± 0.3<br>N: 32.5 ± 0.8	Hypoxic-AT, 60 min of running at 65% VO^{2max}	4 × 3	Normobaric,<br>FiO_2 = 15.0%	Intermittent,<br>60 min/session	④⑤⑫⑬⑭⑮⑯
Morishima et al., 2014	Japan	H: 9 (9/0)<br>N:11 (11/0)	H: 30.0 ± 2.0<br>N: 32.0 ± 3.0	H: 25.6 ± 1.2<br>N: 25.4 ± 0.9	Hypoxic-AT, 60 min cycling at 55% of the maximal oxygen uptake	4 × 3	Normobaric,<br>FiO_2 = 15.0%	Intermittent,<br>60 min/session	①②③⑦⑧⑨⑩⑪⑫
Yang et al., 2014	China	H: 10 (10/0)<br>N: 8 (8/0)	H: 22.5 ± 1.3<br>N: 22.1 ± 2.2	H: 30.7 ± 4.3<br>N: 28.4 ± 2.8	Hypoxic-AT, 60 min cycling and running at 140-150 beats/min	4 × 5	Normobaric,<br>FiO_2 = 15.0%	Intermittent,<br>60 min/session	①②③⑦⑧⑪
Gatterer et al., 2015	Austria	H: 16 (4/12)<br>N: 16 (6/10)	H: 50.3 ± 10.3<br>N: 52.4 ± 7.9	H: 37.9 ± 8.1<br>N: 36.3 ± 4.0	Hypoxic-AT, 90 min of cycling, running and cross trainer at 65-70% HR_{max} and rested for additional 90 min in normobaric hypoxic chambers	32 × 2	Normobaric,<br>FiO_2 = 14.0 ± 0.2%	Intermittent,<br>90 min/session	①②⑤⑥⑦⑧⑪⑭⑮⑯
Zhao et al., 2016	China	H: 9 (9/0)<br>N: 9 (9/0)	H: 18.2 ± 2.2<br>N: 18.1 ± 1.7	H: 32. 9<br>N: 31. 5	Hypoxic-AT, 60 min of cycling at 65-75% VO_{2max}	8 × 5	Normobaric,<br>FiO_2 = 14.7%	Intermittent,<br>60 min/session	①
Park et al., 2017 a	South Korea	H: 11 (0/11)<br>N: 12 (0/12)	H: 42.0 ± 4.4<br>N: 47.2 ± 6.3	H: > 30<br>N: > 30	Hypoxic-AT, 60 min of cycling and running at 75% HR_{max}	6 × 5	Normobaric,<br>FiO_2 = 16.5%	Intermittent,<br>60 min/session	①③⑦⑨⑩⑭⑮
Park et al., 2017 b	South Korea	H: 12 (0/12)<br>N: 12 (0/12)	H: 46.6 ± 5.7<br>N: 47.2 ± 6.3	H: > 30<br>N: > 30	Hypoxic-AT, 60 min of cycling and running at 75% HR_{max}	6 × 5	Normobaric,<br>FiO_2 = 14.5%	Intermittent,<br>60 min/session	①③⑦⑨⑩⑭⑮

Study	Country	Characteristics of participants			Interventions details				Outcomes
		Sample size (M/F)	Age (years)	BMI	Hypoxic training protocol	Period and frequency (week × [times/week])	Type and severity of hypoxia	Pattern and duration of hypoxic exposure	
Shin et al., 2018	Japan	H: 8 (8/0)<br>N: 9 (9/0)	H: 45.6 ± 20.9<br>N: 46.0 ± 20.5	H: 26.8 ± 2.3<br>N: 27.0 ± 3.0	Hypoxic-AT, running in treadmill at 60% HR_{max}, 50 min (5 min warm-up; 40 min main set and 5 min cold down)	4 × 3	Normobaric,<br>FiO_2 = 15.4%	Intermittent,<br>50 min/session	①③⑤⑧⑨⑩⑪⑫⑬
klug et al., 2018	Germany	H: 12 (12/0)<br>N: 11 (11/0)	H: 55.0 ± 7.3<br>N: 57.6 ± 7.3	H: 35.5 ± 4.8<br>N: 34.1 ± 3.0	Hypoxic-AT, 60 min with 3 x 15 min of walking on a treadmill with 5 min of rest	6 × 3	Normobaric,<br>FiO_2 = 15.0%	Intermittent,<br>60 min/session	①②④⑤⑥⑧⑪⑫⑭⑮⑯
Fernández Menéndez et al., 2018	Switzerland	H: 12 (2/10)<br>N: 11 (2/9)	H: 34.8 ± 4.7<br>N: 32.2 ± 8.4	H: 34.0 ± 2.6<br>N: 32.9 ± 2.7	Hypoxic-AT, walking 60 min at six different speeds	3 × 3	Normobaric,<br>FiO_2 = 14.5%	Intermittent,<br>60 min/session	①②④⑦⑧⑨⑩⑪⑫⑬
Zhang et al., 2019	China	H: 20 (20/0)<br>N: 20 (20/0)	H: 22.3 ± 2.2<br>N: 21.9 ± 2.3	H: 32.8 ± 1.2<br>N: 33.3 ± 2.1	Hypoxic-AT, 60 min of cycling at 65% VO_{2max}	4 × 5	Normobaric,<br>FiO_2 = 15.0%	Intermittent,<br>60 min/session	①②⑦⑧⑨⑩⑯
chacaroun et al., 2020	France	H: 12 (11/1)<br>N: 11 (8/3)	H: 52.0 ± 12.0<br>N: 56.0 ± 11.0	H: 31.2 ± 2.4<br>N: 31.8 ± 3.2	Hypoxic-AT, 45 min of cycling at 75% HR_{max}	8 × 3	Normobaric,<br>FiO_2 = 13%	Intermittent,<br>45 min/session	②④⑤⑦⑧⑫⑬⑭⑮⑯
Namboonlue et al., 2021	Thailand	H: 10 (10/0)<br>N: 10 (10/0)	H: 20.3 ± 0.9<br>N: 19.8 ± 0.4	H: 26.3 ± 3.4<br>N: 27.0 ± 1.9	Hypoxic-AT, running at 60% HRR, 30 min (5 min warm-up; 20 min main set and 5 min cold down)	5 × 3	Normobaric,<br>FiO_2 = 15.8%	Intermittent,<br>30 min/session	②③④⑯
Zhang et al., 2018	China	H: 12 (12/0)<br>N: 10 (10/0)	H: 23.8 ± 2.9<br>N: 22.4 ± 1.7	H: 27.2 ± 1.8<br>N: 28.9 ± 1.2	Hypoxic-RT, 3 sets × 10 reps (rest 180s), 70% 1RM Bench press, Squat, Inclined plate horizontal pull, Shoulder press, Deadlift, Standing Elbow flexion	6 × 2	Normobaric,<br>FiO_2 = 15.4%	Intermittent,<br>50 min/session	①③④⑦⑧

Study	Country	Characteristics of participants			Interventions details				Outcomes
		Sample size (M/F)	Age (years)	BMI	Hypoxic training protocol	Period and frequency (week × [times/week])	Type and severity of hypoxia	Pattern and duration of hypoxic exposure	
Li et al., 2019	China	H: 8 (8/0)<br>N: 7 (7/0)	NA	H: 26.5 ± 1.9<br>N: 26.7 ± 1.4	Hypoxic-RT, 3 - 4 sets × 25 reps (rest 120s), 30% 1RM Bench press, Lunge, Deadlift, Push up, Back squat, Plank	6 × 3	Normobaric,<br>FiO_2 = 13.5%	Intermittent,<br>60 min/session	①②③⑦⑧⑨⑯
Torpel et al., 2020	Germany	H: 19 (9/10)<br>N: 17 (9/8)	H: 68.1 ± 4.6<br>N: 67.8 ± 4.1	H: 27.6 ± 4.2<br>N: 26.9 ± 3.6	Hypoxic-RT, 3 sets × 15 reps (rest 30s), 25–40% 1RM 2 training plans with 8 machine-based resistance exercises	5 × 4	Normobaric,<br>SpO_2 = 80–85%	Intermittent,<br>180 min/session	④⑯
Timon et al., 2021	Spain	H: 17<br>N: 18	H: 68.5 ± 3.8<br>N: 70.4 ± 3.4	H: 26.4 ± 3.3<br>N: 27.1 ± 3.9	Hypoxic-RT, 3 sets × 12 reps (rest 60s), 6 - 8 RPE Chest press, Back row, Biceps curl, Triceps push down, Standing lateral raise, Front shoulder raise, Squat, Lying hip raise, Front plank	24 × 3	Normobaric,<br>FiO_2 = 16.1%	Intermittent,<br>45 min/session	④
Namboonlue et al., 2022, a	Thailand	H: 10 (10/0)	H: 20.4 ± 1.2	H: 27.9 ± 4.0	Hypoxic-RT, 3 sets × 15 reps (rest 60s), 50% 1RM knee extension and flexion training	5 × 3	Normobaric,<br>FiO_2 = 15.8%	Intermittent,<br>18 min/session	①②③④
Wang et al., 2012	China	H: 11 (6/5)<br>N: 7 (4/3)	H: 19.5 ± 1.6<br>N: 22.4 ± 2.1	H: 34.6 ± 5.1<br>N: 35.2 ±5.1	Hypoxic-CT, 60 - 70% HR_{max}, 90 min pedaling, stepping, running; 5 sets × 12 reps, 30 min dumbbell exercises, strength training, mat-based training	4 × 3	Normobaric,<br>FiO_2 = 15.4% - 14.8%	Intermittent,<br>120 min/session	①②③⑤⑦⑧⑮
Schreuder et al., 2014	Netherlands	H: 10 (9/1)<br>N: 9 (5/4)	H: 57.0 ± 6.0<br>N: 52.0 ± 8.0	H: 30.9 ± 4.1<br>N: 36.0 ± 6.5	Hypoxic-CT, 70-75% $HR_{reserve}$, 45 min cycling; series of strength training exercises	8 × 3	Normobaric,<br>FiO_2 = 16.5%	Intermittent,<br>NA	①②③⑦⑧⑪⑫⑬⑭⑮⑯

Study	Country	Characteristics of participants: Sample size (M/F)	Age (years)	BMI	Interventions details: Hypoxic training protocol	Period and frequency (week × [times/week])	Type and severity of hypoxia	Pattern and duration of hypoxic exposure	Outcomes
Kong et al., 2014	China	H: 10 (5/5)<br>N: 8 (5/3)	H: 19.8 ± 2.2<br>N: 22.3 ± 1.7	H: 33.8 ± 5.6<br>N: 34.7 ± 5.3	Hypoxic-CT, 60–70% HR_{max}, 90 min running, cycling, stepping; 3 sets × 15 - 20 reps, 40 - 50% 1RM (Rest 2 - 3 min), 30 min 4 - 6 motions	4 × 3	Normobaric, FiO_2 = 16.4% - 14.5%	Intermittent, 120 min/session	①②⑥⑬⑭
González-Muniesa et al., 2015	Spain	H: 14 (14/0)<br>N: 12 (12/0)	H: 25 - 50<br>N: 25 - 50	H: 34.4 ± 2.8<br>N: 33.7 ± 2.7	Hypoxic-CT, 30 min cycling; 4 sets × 15 reps, 30 min 4 Kg weights	13 × 2	Normobaric, FiO_2 = 16.7% - 13.7%	Intermittent, 60 min/session	①②④⑤⑦⑧⑬⑭⑯
Groot et al., 2018	Belgium	H: 7 (3/4)<br>N: 7 (3/4)	H: 12 - 15<br>N: 12 - 15	H: 38.7 ± 1.3<br>N: 36.1 ± 0.9	Hypoxic-CT, 50%-80% MAP, 12 min cycling; 4 sets × 6 reps, 50% - 70%1RM (Rest 120 s), 38-48min abdominal, quadriceps, biceps	6 × 3	Normobaric, FiO_2 = 15%	Intermittent, 50 - 60 min/session	①②④⑦⑧⑪⑫⑬⑭⑮⑯
Park et al., 2019	South Korea	H: 12 (12/0)<br>N: 12 (12/0)	H: 66.5 ± 0.9<br>N: 66.5 ± 0.7	H: 26.0 ± 0.6<br>N: 25.6 ± 0.4	Hypoxic-CT, 60% - 70% HR_{max}, 60 min running, cycling; RT: 3 sets × 10 - 15 reps, 60% - 70% 1RM (Rest 90 s), 30-40min front squat, incline chest press, seated row, push press, split squat, pull apart	12 × 3	Normobaric, FiO_2 = 14.5%	Intermittent, 90 - 120 min/session	①③④
Lian et al., 2020	China	H: 40 (20/20)<br>N: 40 (20/20)	H: 33.1 ± 6.8<br>N: 33.1 ± 6.8	H: 29.8 ±3.9<br>N: 29.2±3.9	Hypoxic-CT, 30 min running; 8 sets × 12 - 15 reps, 12RM (Rest 30 s) 30 min deadlift, rowing, squat, shoulder press, jump, lunge, biceps curl and triceps extension.	6 × 3	Normobaric, FiO_2 = 16%	Intermittent, 60 min/session	①②⑦⑧⑨⑩⑮

Study	Country	Characteristics of participants			Interventions details				Outcomes
		Sample size (M/F)	Age (years)	BMI	Hypoxic training protocol	Period and frequency (week × [times/week])	Type and severity of hypoxia	Pattern and duration of hypoxic exposure	
Wróbel., 2021	Poland	H: 8 (8/0)<br>N: 8 (8/0)	H: 38.8 ± 4.3<br>N: 37.5 ± 5.3	H: 27.1 ± 2.7<br>N: 28.2 ± 1.5	Hypoxic-CT, 50 - 70% HR_{max}, 10 min cycling; 1 sets × 10 reps, barbell bench press and front raise	6 × 3	Normobaric, FiO_2 = 15.4%	Intermittent, 60 min/session	①②③⑥⑯
Namboonlue et al., 2022, b	Thailand	H: 10 (10/0)	H: 21.1 ± 0.6	H: 28.7 ± 4.6	Hypoxic-CT, running at 60% HRR, 30 min (5 min warm-up; 20 min main set and 5 min cold down); 3 sets × 15 reps (rest 60s), 50% 1RM knee extension and flexion training	5 × 3	Normobaric, FiO_2 = 15.8%	Intermittent, 48 min/session	①②③④
Kindlovits., 2024	Portugal	H: 14<br>N: 14	H: 72.2 ± 4.0<br>N: 72.2 ± 4.0	H: 28.3 ± 4.0<br>N: 29.4 ± 4.1	Hypoxic-CT, 40 min cycle ergometer and a treadmill; 3 sets × 10 - 15 reps, 15min Strength exercises with body weight or dumbbells	8 × 3	Normobaric, FiO_2 = 14.7%	Intermittent, 60 min/session	①②③④⑤⑥⑪⑮⑯
Park et al., 2024	South Korea	H: 12 (0/12)<br>N: 12 (0/12)	H: 68.0 ± 1.0<br>N: 68.0 ± 1.0	H: 27.3 ± 0.7<br>N: 27.3 ± 0.7	Hypoxic-CT, 60% - 70% HR_{max}, 60 min running, cycling; 3 sets × 10 - 15 reps, 70 - 80% 1RM (Rest 90 s), 35min bent-over dumbbell deadlift, bent-over dumbbell row, goblet squat	12 × 3	Normobaric, FiO_2 = 14.5%	Intermittent, 120 min/session	①②③④⑬⑭
Kong et al., 2017	China	H: 11 (0/11)<br>N: 13 (0/13)	H: 18 - 30<br>N: 18 - 30	H: 26.0 ± 2.4<br>N: 25.7 ± 2.2	Hypoxic-HIIT, 8s cycling at maximum intensity separated by 12s active recovery at 20- 30 rpm, 60 repetitions.	5 × 4	Normobaric, FiO_2 = 15%	Intermittent, 20 min/session	①②③④⑦⑧⑨⑩⑯

		Characteristics of participants			Interventions details				
Study	Country	Sample size (M/F)	Age (years)	BMI	Hypoxic training protocol	Period and frequency (week × [times/week])	Type and severity of hypoxia	Pattern and duration of hypoxic exposure	Outcomes
Camacho-Cardenosa et al., 2018 a	Spain	H: 15 (0/15)<br>N: 18 (0/18)	H: 37.4 ± 10.3<br>N: 40.1 ± 8.7	H: 27.7 ± 4.6<br>N: 28.7 ± 4.8	Hypoxic-HIIT, 30 s of all-out at 130% W_{max} 8s cycling at maximum intensity separated by 12s active recovery at 20- 30 rpm.	12 × 3	Normobaric, FiO_2 = 17.2%	Intermittent, 24 - 42 min/session	⑤⑥⑦⑧⑪⑬⑭
Camacho-Cardenosa et al., 2018 b	Spain	H: 13 (0/13)<br>N: 15 (0/15)	H: 44.4 ± 7.2<br>N: 43.1 ± 7.7	H: 30.0 ± 6.4<br>N: 29.6 ± 5.3	Hypoxic-HIIT, 3 min cycling at 90% W_{max} separated by 3 min active recovery at 55%–65% W_{max}.	12 × 3	Normobaric, FiO_2 = 17.2%	Intermittent, 16.5 - 27 min/session	⑤⑥⑦⑧⑪⑬⑭
Zheng et al.,2020	China	H: 10 (0/10)<br>N: 10 (0/10)	H: 19.6 ± 1.3<br>N: 19.6 ± 1.3	H: > 30%<br>N: > 30%	Hypoxic-HIIT, 10 min running separated by 3 min rest, 3 - 5 repetitions.	6 × 3	Normobaric, FiO_2 = 14.4%	Intermittent, 39 - 65 min/session	①②③
Hobbins et al., 2021	UK	H: 8 (4/4)<br>N: 8 (5/3)	H: 32.1 ± 10.2<br>N: 41.1 ± 13.0	H: 31.9 ± 3.6<br>N: 33.0 ± 1.4	Hypoxic-HIIT, 2 min walking velocity (RPE = 14 separated by 2 min rest,15 repetitions.	2 × 4	Normobaric, FiO_2 = 13%	Intermittent, 60 min/session	①②⑬⑭
Ghaith et al., 2022	France	H: 16 (10/6)<br>N: 15 (13/2)	H: 51.0 ± 8.3<br>N: 52.0 ± 7.5	H: 31.5 ± 4.0<br>N: 32.4 ± 4.8	Hypoxic-HIIT, 30s - 1min cycling at 80% W_{peak} separated by 30s - 1min passive recovery,16-32 repetitions.	8 × 3	Normobaric, FiO_2 = 12%	Intermittent, 16 - 45 min/session	①②④⑤⑦⑧⑪⑫⑬⑭⑮⑯
Baginska et al., 2024	Poland	H: 12 (0/12)<br>N: 12 (0/12)	H: 41.3 ± 10.5<br>N: 41.3 ± 10.5	H: 32.6 ± 6.4<br>N: 30.4 ± 2.0	Hypoxic-HIIT, 6 min cycling at 85% HR_{max} separated by 6 min active recovery cycling at 70% HR_{max}, 5 repetitions.	4 × 3	Normobaric, FiO_2 = 15.4%	Intermittent, 60 min/session	①②④⑯

M male *F* female *N* normoxic group *H* hypoxic group *Hypoxic-AT* hypoxic aerobic training *Hypoxic-RT* hypoxic resistance training *Hypoxic-CT* hypoxic resistance combined with hypoxic aerobic training *Hypoxic-HIIT* hypoxic high-intensity interval training *NA* no available *FiO_2* inspired fraction of oxygen *HR_{max}* maximum heart rate *VO_{2max}* maximal oxygen consumption *HRR* heart rate reserve *Intermittent* hypoxic training consisting of hypoxia exposure lasting seconds to hours with a return to normoxia or

lower levels of hypoxia and repetition over days to weeks ① body mass ② body mass index ③ fat mass ④ fat-free mass ⑤ waist circumference ⑥ waist-to-hip ratio ⑦ total cholesterol ⑧ triglycerides ⑨ high-density lipoprotein cholesterol ⑩ low-density lipoprotein cholesterol ⑪ glucose ⑫ insulin ⑬ homeostasis model assessment of insulin resistance ⑭ systolic blood pressure ⑮ diastolic blood pressure ⑯ maximal oxygen consumption.

Supplement 3: Risk of bias

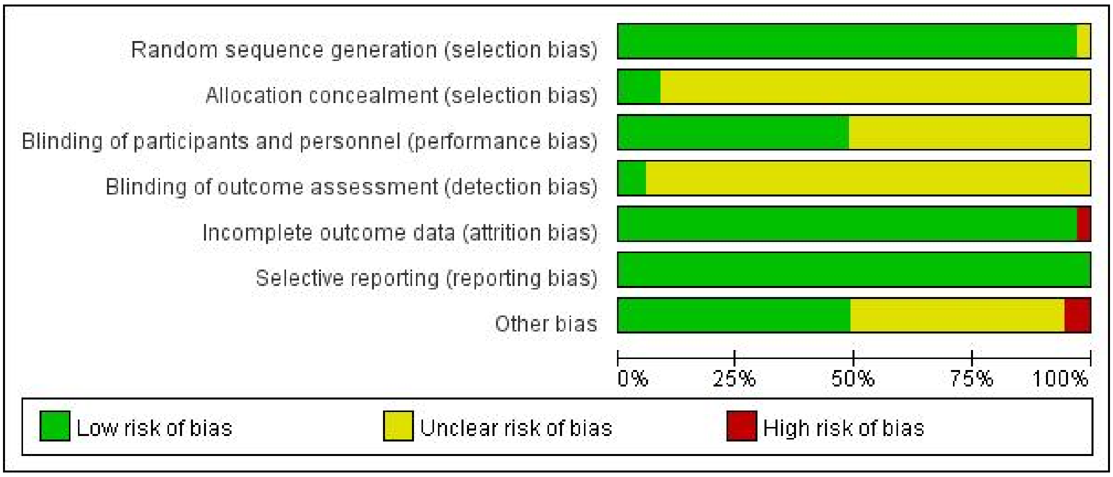

Fig. S1 The overall risk of bias for all included studies

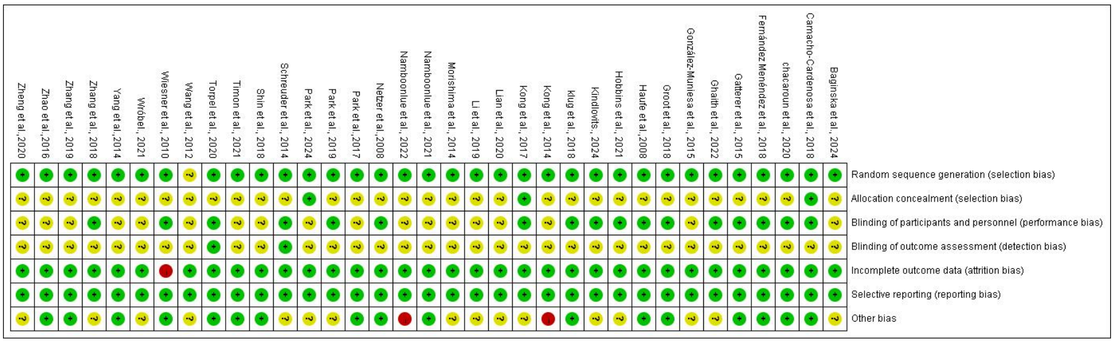

Fig. S2 The risk of bias for each study

Supplement 4: Forest plots

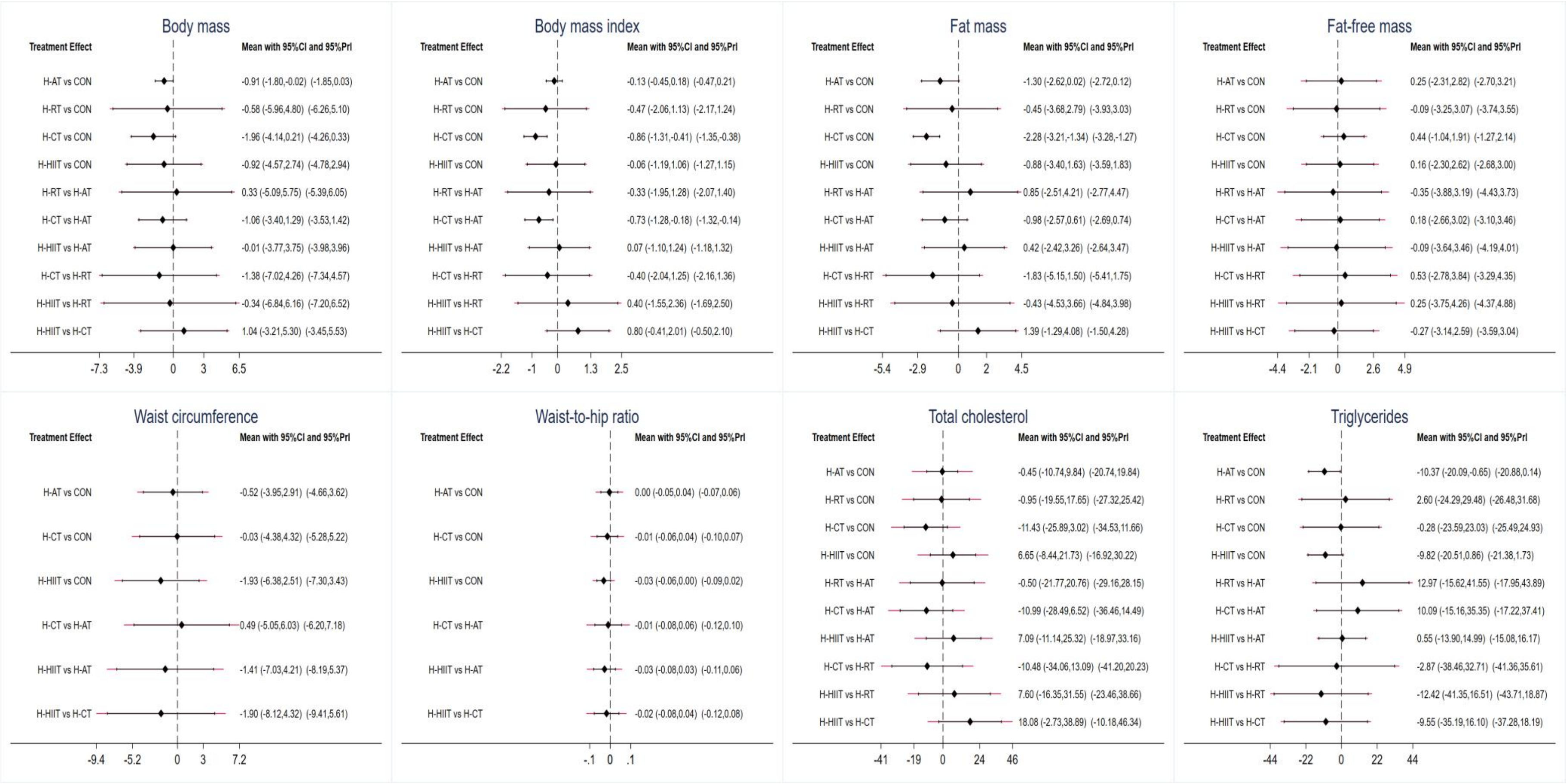

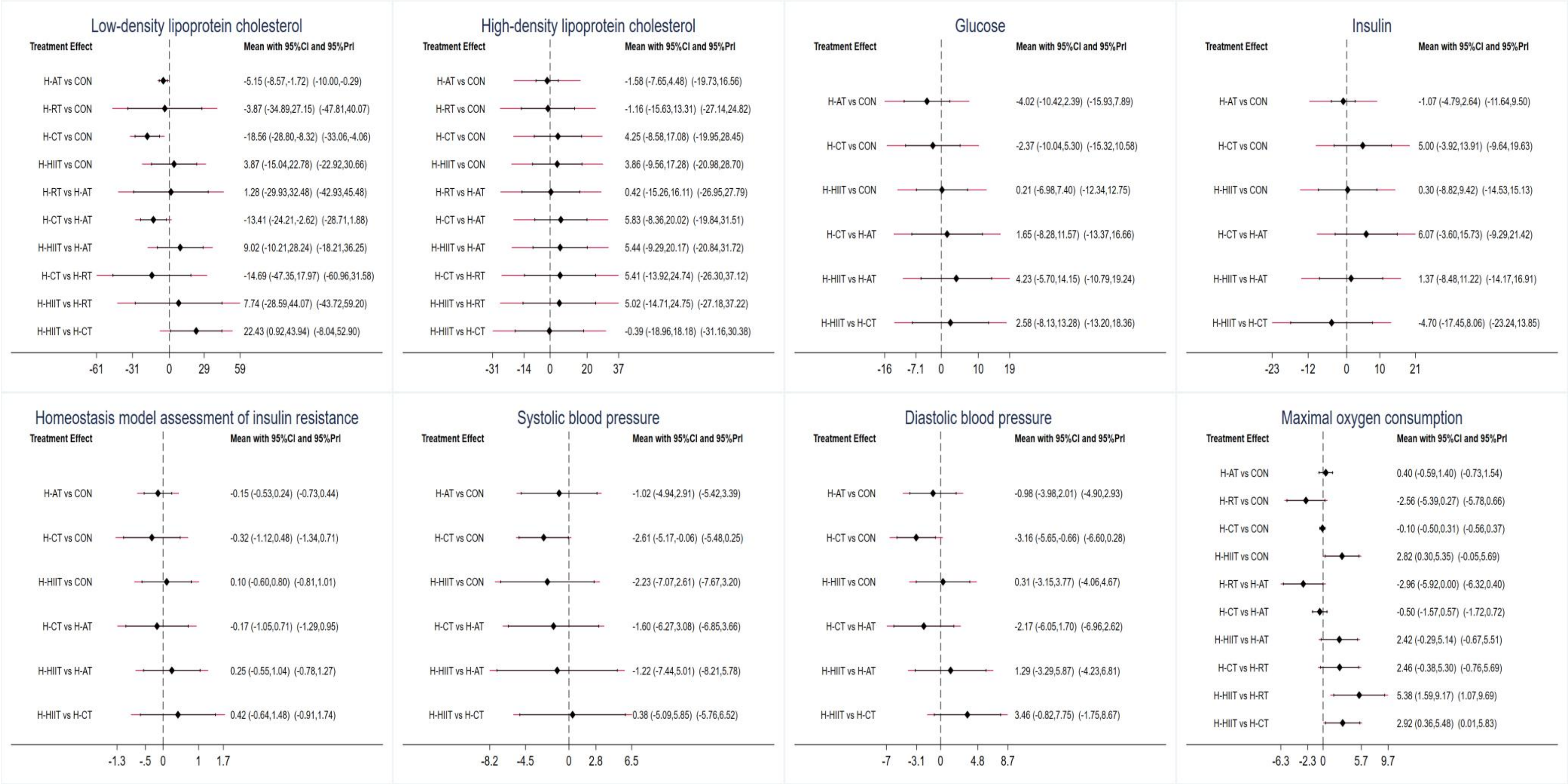

CON Control *Hypoxic-AT* hypoxic aerobic training *Hypoxic-RT* hypoxic resistance training *Hypoxic-CT* hypoxic resistance combined with hypoxic aerobic training *Hypoxic-HIIT* hypoxic high-intensity interval training.

Supplement 5: Heterogeneity assessments

Outcomes	Design-based Q statistic, comparisons, and overall statement	Q statistic	Degree of freedom	*P* -value
**Body mass**	**Design-specific decomposition of within-designs Q statistic**			
	Control vs. Hypoxic-RT	0.03	1	0.8638
	Control vs. Hypoxic-AT	3.62	12	0.9894
	Control vs. Hypoxic-HIIT	0.23	4	0.9937
	Control vs. Hypoxic-CT	1.19	9	0.9989
	**Between-designs Q statistic after detaching of single designs**			
	Control vs. Hypoxic-CT	0.03	1	0.8617
	Control vs. Hypoxic-AT	0.03	1	0.8560
	Control vs. Hypoxic-RT	0.15	1	0.6994
	Q statistic to assess consistency under the assumption of a full design-by-treatment interaction random effects model: between designs Q statistic, 0.15; degree of freedom, 2; *p*-value, 0.9281; tau. within, 0; tau^2.within, 0.			
**Body mass Index**	**Design-specific decomposition of within-designs Q statistic**			
	Control vs. Hypoxic-AT	7.34	9	0.6015

	Control vs. Hypoxic-HIIT	2.07	4	0.7224
	Control vs. Hypoxic-CT	2.98	8	0.9358
	**Between-designs Q statistic after detaching of single designs**			
	Control vs. Hypoxic-AT	0.00	1	0.9521
	Control vs. Hypoxic-CT	0.45	1	0.5034
	Control vs. Hypoxic-RT	0.48	1	0.4892
	Q statistic to assess consistency under the assumption of a full design-by-treatment interaction random effects model: between designs Q statistic, 0.68; degree of freedom, 2; p-value, 0.7126; tau. within, 0; tau^2.within, 0.			
**Fat mass**	**Design-specific decomposition of within-designs Q statistic**			
	Control vs. Hypoxic-AT	7.34	9	0.6015
	Control vs. Hypoxic-HIIT	2.07	4	0.7224
	Control vs. Hypoxic-CT	2.98	8	0.9358
	**Between-designs Q statistic after detaching of single designs**			
	Control vs. Hypoxic-AT	0.00	1	0.9521
	Control vs. Hypoxic-CT	0.45	1	0.5034

	Control vs. Hypoxic-RT	0.48	1	0.4892
	Q statistic to assess consistency under the assumption of a full design-by-treatment interaction random effects model: between designs Q statistic, 0.68; degree of freedom, 2; *p*-value, 0.7126; tau. within, 0; tau^2.within, 0.			
**Fat-free mass**	**Design-specific decomposition of within-designs Q statistic**			
	Control vs. Hypoxic-AT	2.14	4	0.7103
	Control vs. Hypoxic-HIIT	0.20	2	0.9063
	Control vs. Hypoxic-RT	0.07	2	0.9668
	Control vs. Hypoxic-AT	0.12	4	0.9983
	**Between-designs Q statistic after detaching of single designs**			
	Control vs. Hypoxic-CT	0.04	1	0.8372
	Control vs. Hypoxic-AT	0.15	1	0.7029
	Control vs. Hypoxic-RT	0.40	1	0.5282
	Q statistic to assess consistency under the assumption of a full design-by-treatment interaction random effects model: between designs Q statistic, 0.41; degree of freedom, 2; p-value, 0.8144; tau. within, 0; tau^2.within, 0.			
**Waist circumference**	**Design-specific decomposition of within-designs Q statistic**			
	Control vs. Hypoxic-HIIT	1.38	2	0.5013

	Control vs. Hypoxic-CT	0.17	2	0.9202
	Control vs. Hypoxic-AT	0.33	4	0.9881
	Q statistic to assess consistency under the assumption of a full design-by-treatment interaction random effects model: between designs Q statistic, 0.00; degree of freedom, 0; p-value, --; tau. within, 0; tau^2.within, 0.			
**Waist-to-hip ratio**	**Design-specific decomposition of within-designs Q statistic**			
	Control vs. Hypoxic-HIIT	0.75	1	0.3861
	Control vs. Hypoxic-AT	0.05	1	0.8182
	Control vs. Hypoxic-CT	0.34	2	0.8430
	Q statistic to assess consistency under the assumption of a full design-by-treatment interaction random effects model: between designs Q statistic, 0.00; degree of freedom, 0; p-value, --; tau. within, 0; tau^2.within, 0.			
**Total cholesterol**	**Design-specific decomposition of within-designs Q statistic**			
	Control vs. Hypoxic-CT	10.70	4	0.0301
	Control vs. Hypoxic-AT	7.13	8	0.5232
	Control vs. Hypoxic-HIIT	1.33	3	0.7221
	Control vs. Hypoxic-RT	0.00	1	0.9691
	Q statistic to assess consistency under the assumption of a full design-by-treatment interaction random effects model: between designs Q			

	statistic, 0.00; degree of freedom, 0; p-value, --; tau. within, 5.9209; tau^2.within, 35.0576.			
**Triglycerides**	**Design-specific decomposition of within-designs Q statistic**			
	Control vs. Hypoxic-CT	8.14	4	0.0865
	Control vs. Hypoxic-RT	0.46	1	0.4987
	Control vs. Hypoxic-AT	7.58	9	0.5766
	Control vs. Hypoxic-HIIT	1.29	3	0.7303
	Q statistic to assess consistency under the assumption of a full design-by-treatment interaction random effects model: between designs Q statistic, 0.00; degree of freedom, 0; p-value, --; tau. within, 3.1012; tau^2.within, 9.6176.			
**Low-density lipoprotein cholesterol**	**Design-specific decomposition of within-designs Q statistic**			
	Control vs. Hypoxic-AT	0.88	5	0.9716
	Q statistic to assess consistency under the assumption of a full design-by-treatment interaction random effects model: between designs Q statistic, 0.00; degree of freedom, 0; p-value, --; tau. within, 0; tau^2.within, 0.			
**High-density lipoprotein cholesterol**	**Design-specific decomposition of within-designs Q statistic**			
	Control vs. Hypoxic-AT	13.18	5	0.0218
	Q statistic to assess consistency under the assumption of a full design-by-treatment interaction random effects model: between designs Q statistic, 0.00; degree of freedom, 0; p-value, --; tau. within, 5.8409; tau^2.within, 34.1157.			

**Glucose**	**Design-specific decomposition of within-designs Q statistic**			
	Control vs. Hypoxic-HIIT	7.33	2	0.0256
	Control vs. Hypoxic-AT	2.03	4	0.7310
	Control vs. Hypoxic-CT	0.36	2	0.8368
	Q statistic to assess consistency under the assumption of a full design-by-treatment interaction random effects model: between designs Q statistic, 0.00; degree of freedom, 0; p-value, --; tau. within, 2.5661; tau^2.within, 6.5848.			
**Insulin**	**Design-specific decomposition of within-designs Q statistic**			
	Control vs. Hypoxic-AT	15.83	6	0.0147
	Control vs. Hypoxic-CT	0.00	1	0.9703
	Q statistic to assess consistency under the assumption of a full design-by-treatment interaction random effects model: between designs Q statistic, 0.00; degree of freedom, 0; p-value, --; tau. within, 3.1970; tau^2.within, 10.2207.			
**Homeostasis model assessment of insulin resistance**	**Design-specific decomposition of within-designs Q statistic**			
	Control vs. Hypoxic-AT	15.83	6	0.0147
	Control vs. Hypoxic-CT	0.0	1	0.9703
	Q statistic to assess consistency under the assumption of a full design-by-treatment interaction random effects model: between designs Q statistic, 0.00; degree of freedom, 0; p-value, --; tau. within, 3.1970; tau^2.within, 10.2207.			

**Systolic blood pressure**	**Design-specific decomposition of within-designs Q statistic**			
	Control vs. Hypoxic-HIIT	3.17	3	0.3664
	Control vs. Hypoxic-AT	4.03	5	0.5444
	Control vs. Hypoxic-CT	2.98	4	0.5616
	Q statistic to assess consistency under the assumption of a full design-by-treatment interaction random effects model: between designs Q statistic, 0.00; degree of freedom, 0; p-value, --; tau. within, 0; tau^2.within, 0.			
**Diastolic blood pressure**	**Design-specific decomposition of within-designs Q statistic**			
	Control vs. Hypoxic-CT	4.72	4	0.3168
	Control vs. Hypoxic-AT	4.18	5	0.5235
	Control vs. Hypoxic-HIIT	0.79	3	0.8516
	Q statistic to assess consistency under the assumption of a full design-by-treatment interaction random effects model: between designs Q statistic, 0.00; degree of freedom, 0; p-value, --; tau. within, 0; tau^2.within, 0.			
**Maximal oxygen consumption**	**Design-specific decomposition of within-designs Q statistic**			
	Control vs. Hypoxic-HIIT	1.13	2	0.5674
	Control vs. Hypoxic-AT	4.30	6	0.6360
	Control vs. Hypoxic-CT	2.06	4	0.7242

	Control vs. Hypoxic-RT	0.01	1	0.9046
	**Between-designs Q statistic after detaching of single designs**			
	Control vs. Hypoxic-RT	0.00	1	0.9720
	Control vs. Hypoxic-CT	0.89	1	0.3459
	Control vs. Hypoxic-AT	1015	1	0.2830
	Q statistic to assess consistency under the assumption of a full design-by-treatment interaction random effects model: between designs Q statistic, 1.23; degree of freedom, 2; *p*-value, 0.5413; tau. within, 0; tau^2.within, 0.			

Hypoxic-AT hypoxic aerobic training *Hypoxic-RT* hypoxic resistance training *Hypoxic-CT* hypoxic resistance combined with hypoxic aerobic training *Hypoxic-HIIT* hypoxic high-intensity interval training.

Supplement 6: Intransitivity assessments

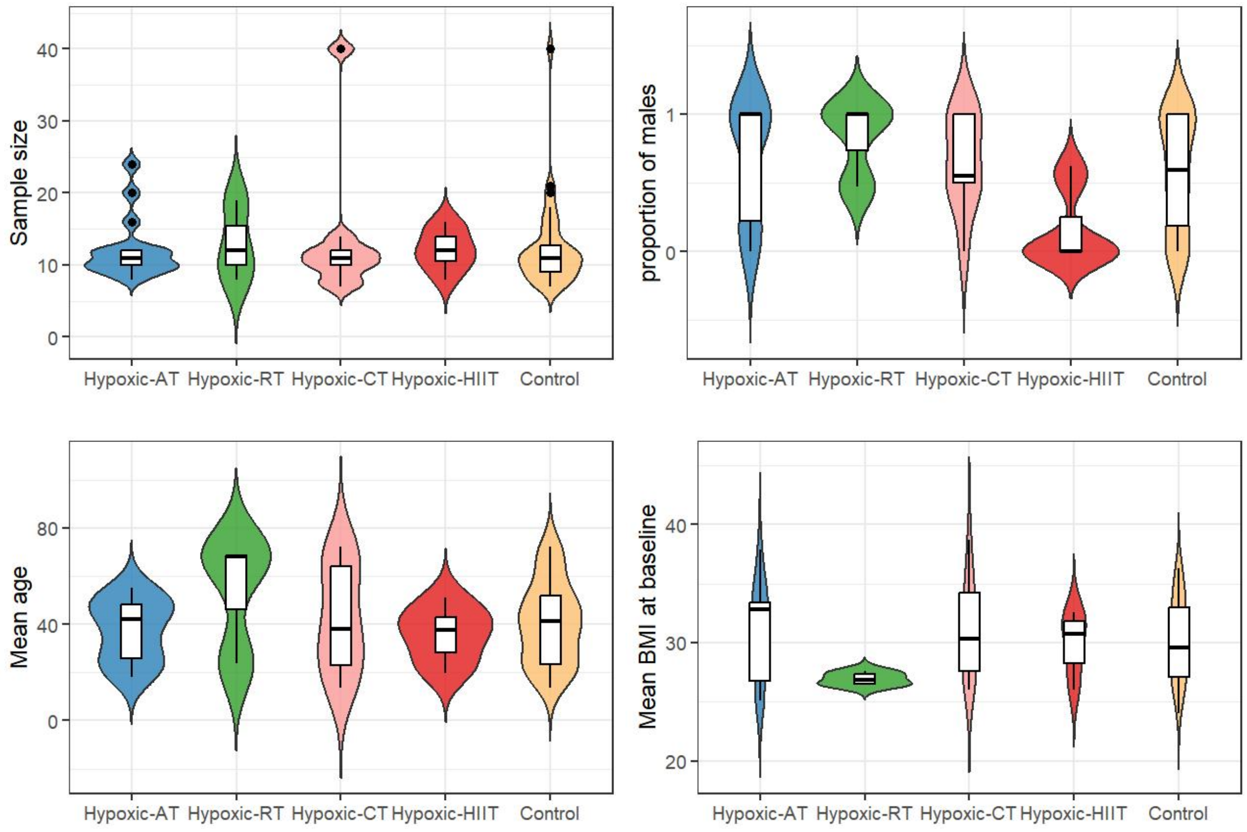

Hypoxic-AT hypoxic aerobic training *Hypoxic-RT* hypoxic resistance training *Hypoxic-CT* hypoxic resistance combined with hypoxic aerobic training *Hypoxic-HIIT* hypoxic high-intensity interval training.

Supplement 7: Inconsistency analysis

Comparison	Beta coefficients (standard error)			Significance of difference in estimates (p-value)
	Direct comparison	Indirect comparison via network	Difference in estimates	
**Body mass**				
Control vs. Hypoxic-AT	-0.90 (0.46)	-2.63 (5.11)	1.73 (5.13)	0.737
Control vs. Hypoxic-RT	-0.58 (3.15)	-0.55 (4.39)	-0.02 (5.62)	0.996
Control vs. Hypoxic-CT	-2.02 (1.14)	-0.24 (5.10)	-1.77 (5.23)	0.735
Hypoxic-AT vs. Hypoxic-RT	1.24 (5.15)	-0.05 (3.35)	1.29 (6.23)	0.836
Hypoxic-AT vs. Hypoxic-CT	1.09 (5.70)	-1.77 (3.53)	1.21 (5.83)	0.704
Hypoxic-RT vs. Hypoxic-CT	-0.47 (5.19)	-1.77 (3.53)	1.29 (6.38)	0.839
**Body mass index**				
Control vs. Hypoxic-AT	-0.11 (0.16)	-1.32 (1.46)	1.21 (1.47)	0.421
Control vs. Hypoxic-RT	-0.70 (0.96)	0.09 (1.49)	-0.79 (1.78)	0.655
Control vs. Hypoxic-CT	-0.87 (0.23)	-0.12 (1.54)	-0.75 (1.56)	0.632
Hypoxic-AT vs. Hypoxic-RT	0.74 (1.64)	-0.67 (0.93)	1.41 (1.87)	0.449
Hypoxic-AT vs. Hypoxic-CT	0.44 (1.65)	-0.76 (0.28)	1.21 (1.67)	0.469
Hypoxic-RT vs. Hypoxic-CT	-0.30 (1.77)	-0.42 (0.95)	0.11 (2.01)	0.954
**Fat mass**				
Control vs. Hypoxic-AT	-0.60 (0.57)	-2.73 (2.50)	2.12 (2.56)	0.407
Control vs. Hypoxic-RT	-0.16 (1.97)	-0.67 (2.61)	0.50 (3.27)	0.877
Control vs. Hypoxic-CT	-3.10 (0.39)	-2.88 (2.73)	-2.81 (2.75)	0.307
Hypoxic-AT vs. Hypoxic-RT	1.39 (2.78)	-0.14 (1.95)	1.53 (3.34)	0.645

Hypoxic-AT vs. Hypoxic-CT	0.42 (2.81)	-2.50 (0.69)	2.92 (2.90)	0.314
Hypoxic-RT vs. Hypoxic-CT	-0.62 (3.14)	-3.43 (1.87)	2.80 (3.66)	0.444
**Fat-free mass**				
Control vs. Hypoxic-AT	0.74 (1.62)	-0.91 (2.62)	1.65 (3.08)	0.591
Control vs. Hypoxic-RT	0.08 (2.25)	-0.26 (2.31)	0.34 (3.23)	0.914
Control vs. Hypoxic-CT	0.31 (0.78)	2.14 (2.87)	-1.83 (2.97)	0.537
Hypoxic-AT vs. Hypoxic-RT	0.07 (2.47)	-0.90 (2.74)	0.97 (3.72)	0.793
Hypoxic-AT vs. Hypoxic-CT	1.78 (2.91)	-0.45 (1.77)	2.24 (3.42)	0.513
Hypoxic-RT vs. Hypoxic-CT	1.41 (2.66)	-0.13 (2.27)	1.54 (3.57)	0.667
**Maximal oxygen consumption**				
Control vs. Hypoxic-AT	0.37 (0.52)	1.05 (2.44)	-0.68 (2.49)	0.784
Control vs. Hypoxic-RT	-1.01 (2.00)	-4.22 (2.08)	3.20 (2.89)	0.268
Control vs. Hypoxic-CT	-0.10 (0.20)	1.13 (2.11)	-1.24 (2.13)	0.560
Hypoxic-AT vs. Hypoxic-RT	-4.91 (2.75)	-2.01 (1.87)	-2.89 (3.42)	0.398
Hypoxic-AT vs. Hypoxic-CT	-0.01 (2.44)	-5.22 (0.55)	0.5 (2.49)	0.838
Hypoxic-RT vs. Hypoxic-CT	3.90 (2.15)	1.38 (1.87)	2.52 (2.77)	0.362

Hypoxic-AT hypoxic aerobic training *Hypoxic-RT* hypoxic resistance training *Hypoxic-CT* hypoxic resistance combined with hypoxic aerobic training *Hypoxic-HIIT* hypoxic high-intensity interval training.

Supplement 8: Comparison-adjusted funnel plots

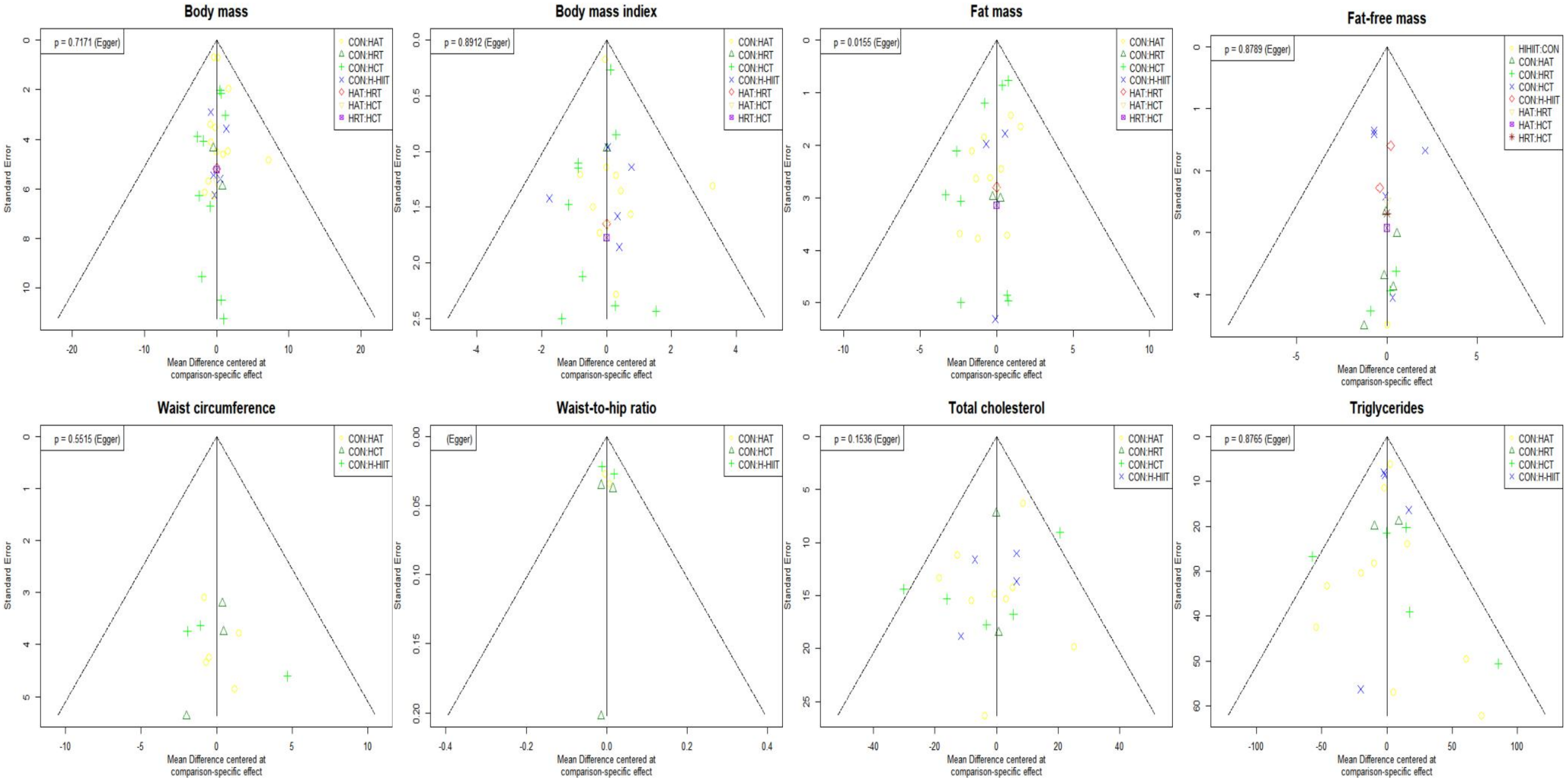

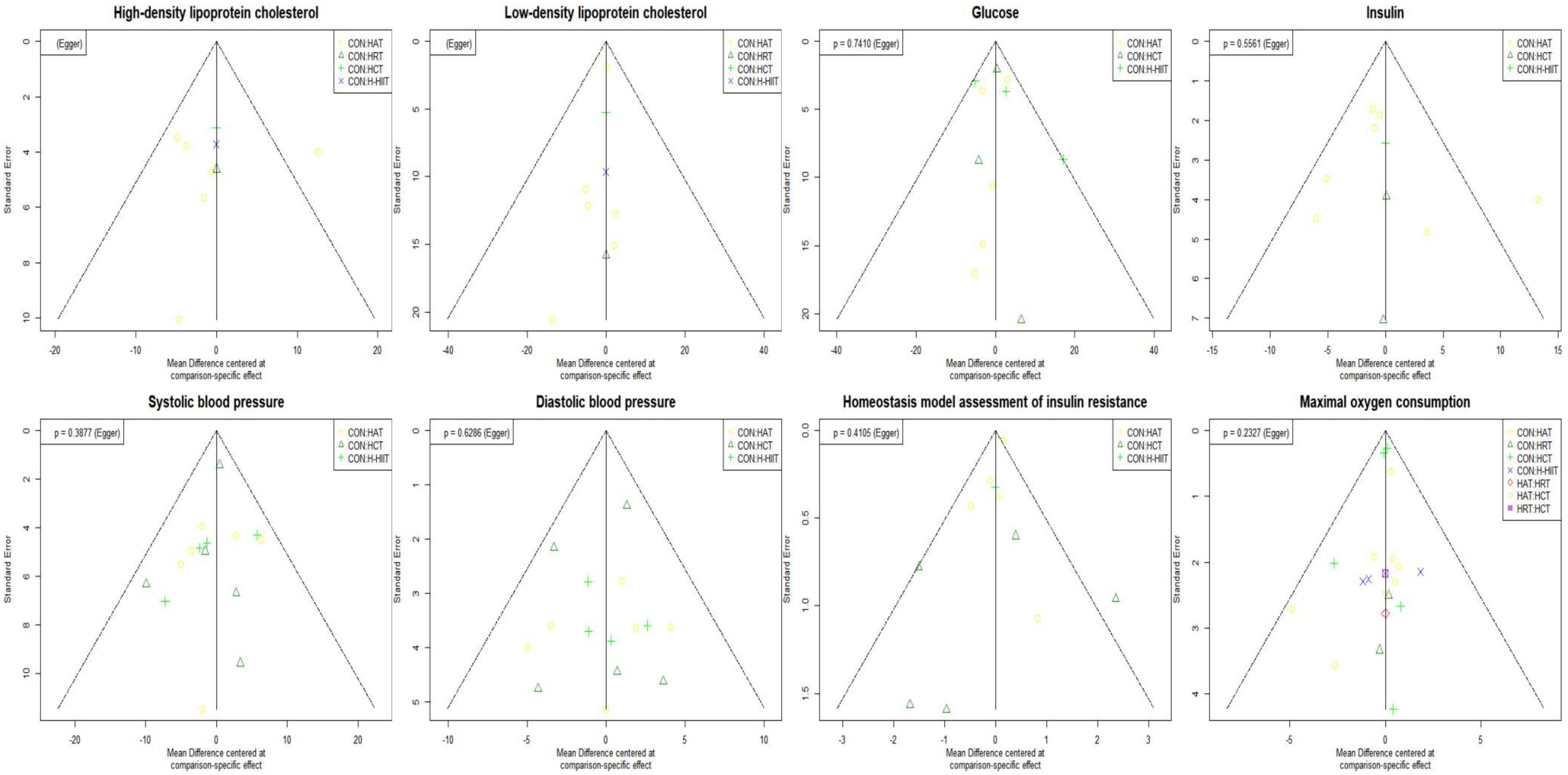

CON control *Hypoxic-AT* hypoxic aerobic training *Hypoxic-RT* hypoxic resistance training *Hypoxic-CT* hypoxic resistance combined with hypoxic aerobic training *Hypoxic-HIIT* hypoxic high-intensity interval training.

Supplement 9: Summary of pairwise meta-analyses

Outcomes	Contrast to normoxic	Studies	n	Forest plot (REM)	MD (95% of CI)	p-value	I2 (p)	Low ROB (%)	Egger's p	GRADE
TC	Hypoxic training	20	525		-1.81 (-8.26, 4.64)	0.58	23% (0.17)	45	0.267	Very low (a,c,d)
	Hypoxic-AT	9	215		-1.93 (-9.84, 5.99)	0.63	0% (0.52)	18	0.485	Very low (a,c,d)
	Hypoxic-RT	2	37		-0.88 (-14.13, 12.37)	0.89	0% (0.97)	83	n/a	n/a
	Hypoxic-CT	5	157		-8.96 (-29.32, 11.41)	0.39	63% (0.03)	28	0.199	Very low (a,b,c,d)
	Hypoxic-HIIT	4	116		6.38 (-6.30, 19.06)	0.32	0% (0.72)	100	0.545	Low (c,d)
TG	Hypoxic training	21	541		-8.87 (-15.51, -2.22)	< 0.01	0% (0.59)	62	0.798	Low (a,c)
	Hypoxic-AT	10	231		-10.37 (-20.09, -0.65)	0.04	0% (0.58)	35	0.822	Low (a,c)
	Hypoxic-RT	2	37		-3.09 (-29.26, 23.08)	0.82	0% (0.82)	49	n/a	n/a
	Hypoxic-CT	5	157		-2.65 (-38.35, 33.05)	0.88	51% (0.08)	45	0.547	Very low (a,b,c,d)
	Hypoxic-HIIT	4	116		-9.82 (-20.51, 0.86)	0.07	0% (0.73)	100	0.807	Low (c,d)
LDL-C	Hypoxic training	9	266		-6.18 (-9.36, -2.99)	< 0.01	0% (0.44)	22	0.744	Low (a,c)
	Hypoxic-AT	6	147		-5.15 (-8.57, -1.72)	< 0.01	0% (0.97)	2	0.221	Low (a,c)
	Hypoxic-RT	1	15		-3.87 (-34.89, 27.15)	0.81	n/a	0	n/a	n/a
	Hypoxic-CT	1	80		-18.56 (-28.80, -8.32)	< 0.01	n/a	0	n/a	n/a
	Hypoxic-HIIT	1	24		3.87 (-15.04, 22.78)	0.69	n/a	100	n/a	n/a
HDL-C	Hypoxic training	9	266		0.02 (-4.07, 4.10)	0.99	53% (0.03)	22	0.665	Very low (a,b,c,d)
	Hypoxic-AT	6	147		-1.58 (-7.70, 4.54)	0.61	62% (0.02)	2	0.943	Very low (a,b,c,d)
	Hypoxic-RT	1	15		-1.16 (-10.22, 7.90)	0.80	n/a	0	n/a	n/a
	Hypoxic-CT	1	80		4.25 (-1.86, 10.36)	0.17	n/a	0	n/a	n/a
	Hypoxic-HIIT	1	24		3.86 (-3.42, 11.14)	0.30	n/a	100	n/a	n/a
Glucose	Hypoxic training	11	263		-2.06 (-5.89, 1.77)	0.29	30% (0.16)	81	0.771	Very low (a,b,c,d)
	Hypoxic-AT	5	110		-4.35 (-9.34, 0.65)	0.09	0% (0.73)	73	0.490	Low (a,c)
	Hypoxic-RT	0								
	Hypoxic-CT	3	61		-2.74 (-6.67, 1.19)	0.17	0% (0.84)	100	0.897	Low (c,d)
	Hypoxic-HIIT	3	92		-1.67 (-12.40, 9.06)	0.76	73% (0.03)	100	0.248	Very low (b,c,d)
Insulin	Hypoxic training	9	218		0.62 (-1.12, 2.36)	0.49	0% (0.72)	80	0.163	Very low (a,c,d)
	Hypoxic-AT	6	154		0.30 (-1.63, 2.24)	0.76	0% (0.60)	68	0.574	Very low (a,c,d)
	Hypoxic-RT	0								
	Hypoxic-CT	2	33		4.97 (-1.74, 11.68)	0.15	0% (0.97)	100	n/a	n/a
	Hypoxic-HIIT	1	31		0.30 (-4.73, 5.33)	0.91	n/a	100	n/a	n/a
HOMA-IR	Hypoxic training	11	318		-0.15 (-0.35, 0.05)	0.13	38% (0.17)	68	0.532	Very low (a,b,c,d)
	Hypoxic-AT	5	128		-0.19 (-0.37, -0.01)	0.04	0% (0.50)	98	0.401	Low (a,c)
	Hypoxic-RT	0								
	Hypoxic-CT	5	159		-0.19 (-1.71, 1.33)	0.81	66% (0.02)	74	0.614	Very low (a,b,c,d)
	Hypoxic-HIIT	1	31		0.10 (-0.53, 0.73)	0.76	n/a	100	n/a	n/a
SBP	Hypoxic training	15	373		-2.15 (-4.11, -0.20)	0.03	0% (0.71)	60	0.220	Low (a,c)
	Hypoxic-AT	6	165		-1.02 (-4.94, 2.91)	0.61	0% (0.54)	58	0.681	Very low (a,c,d)
	Hypoxic-RT	0								
	Hypoxic-CT	5	100		-2.61 (-5.17, -0.06)	0.05	0% (0.56)	3	0.588	Low (a,c)
	Hypoxic-HIIT	4	108		-2.18 (-7.17, 2.81)	0.39	5.3% (0.37)	100	0.217	Low (c,d)
DBP	Hypoxic training	15	373		-1.93 (-3.44, -0.42)	0.01	0% (0.49)	66	0.128	Low (a,c)
	Hypoxic-AT	6	165		-1.00 (-3.90, 1.89)	0.50	0% (0.52)	58	0.642	Very low (a,c,d)
	Hypoxic-RT	0								
	Hypoxic-CT	5	100		-3.09 (-5.63, -0.55)	0.02	15% (0.32)	4	0.714	Low (a,c)
	Hypoxic-HIIT	4	108		0.32 (-3.01, 3.66)	0.85	0% (0.85)	100	0.525	Low (c,d)
VO2max	Hypoxic training	17	385		0.01 (-0.36, 0.38)	0.95	0% (0.65)	59	0.024	Very low (a,c,d,e)
	Hypoxic-AT	7	152		0.37 (-0.65,1.39)	0.47	0% (0.64)	90	0.206	Very low (a,c,d)
	Hypoxic-RT	2	51		-1.02 (-4.95, 2.91)	0.61	0% (0.91)	35	n/a	n/a
	Hypoxic-CT	5	103		-0.11 (-0.52, 0.30)	0.60	0% (0.72)	62	0.519	Very low (a,c,d)
	Hypoxic-HIIT	3	79		2.82 (0.30, 5.35)	0.03	0% (0.57)	68	0.091	Low (a,c)

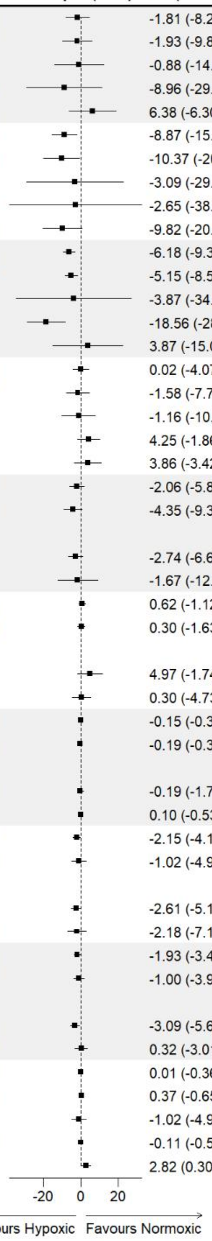

Cardiometabolic health outcomes in pairwise meta-analyses. *Hypoxic-AT* hypoxic aerobic training; *Hypoxic-RT* hypoxic resistance training; *Hypoxic-CT* hypoxic resistance combined with hypoxic aerobic training; *Hypoxic-HIIT* hypoxic high-intensity interval training. *TC* total cholesterol; *TG* triglycerides; *HDL-C* high-density lipoprotein cholesterol; *LDL-C* low-density lipoprotein cholesterol; *HOMA-IR* homeostasis model assessment of insulin resistance; *SBP* systolic blood pressure; *DBP* diastolic blood pressure; VO_{2max} maximal oxygen consumption. *REM* random effects model, *ROB* risk of bias (percentage of studies with low), *MD* mean difference, GRADE certainty ratings *very low* the true effect is probably markedly different from the estimated effect, *low* the true effect might be markedly different from the estimated effect, *moderate* the true effect is probably close to the estimated effect, *high* the true effect is similar to the estimated effect. [a]: down rating for risk of bias, [b]: down rating for inconsistency, [c]: down rating for indirectness, [d]: down rating for imprecision, [e]: down rating for publication bias (using Egger's *p*-value).

Outcomes	Contrast to normoxic	Studies	n	Forest plot (REM)	MD (95% of CI)	p-value	I2 (p)	Low ROB (%)	Egger's p	GRADE
BM	Hypoxic training	30	717		-1.04 (-1.84, -0.24)	0.01	0% (1.00)	41	0.293	Low (a,c)
	Hypoxic-AT	13	298		-0.89 (-1.79, 0.00)	0.05	0% (0.98)	92	0.369	Low (a,c)
	Hypoxic-RT	2	37		-0.59(-7.48, 6.30)	0.87	0% (0.86)	14	n/a	n/a
	Hypoxic-CT	10	267		-2.05 (-4.28, 0.18)	0.07	0% (1.00)	37	0.177	Very low (a,c,d)
	Hypoxic-HIIT	5	115		-0.92 (-4.57, 2.74)	0.62	0% (0.99)	62	0.818	Very low (a,c,d)
BMI	Hypoxic training	25	607		-0.69 (-1.06, -0.32)	< 0.01	0% (0.95)	37	0.672	Low (a,c)
	Hypoxic-AT	10	236		-0.12 (-0.43, 0.20)	0.47	0% (0.69)	92	0.994	Very low (a,c,d)
	Hypoxic-RT	1	15		-0.70 (-2.60, 1.20)	0.47	n/a	9	n/a	n/a
	Hypoxic-CT	9	243		-0.88 (-1.33, -0.42)	< 0.01	0% (0.94)	5	0.103	Low (a,c)
	Hypoxic-HIIT	5	113		-0.06 (-1.19, 1.06)	0.92	0% (0.72)	51	0.803	Very low (a,c,d)
FFM	Hypoxic training	16	411		0.31 (-0.84, 1.46)	0.59	0% (1.00)	62	0.766	Very low (a,c,d)
	Hypoxic-AT	5	122		0.64 (-2.31, 3.60)	0.67	0% (0.99)	57	0.348	Very low (a,c,d)
	Hypoxic-RT	3	94		0.08 (-4.35, 4.52)	0.97	0% (0.97)	45	0.190	Very low (a,c,d)
	Hypoxic-CT	5	116		0.31 (-1.22, 1.84)	0.69	0% (0.71)	47	0.695	Very low (a,c,d)
	Hypoxic-HIIT	3	79		0.16 (-2.30, 2.62)	0.9	0% (0.91)	70	0.586	Very low (a,c,d)
FM	Hypoxic training	24	577		-1.82 (-2.55, -1.09)	< 0.01	0% (0.98)	31	< 0.001	Very low (a,c,e)
	Hypoxic-AT	10	224		-1.22 (-2.59, 0.15)	0.08	0% (1.00)	22	0.088	Very low (a,c,d)
	Hypoxic-RT	2	37		-0.25 (-4.40, 3.90)	0.91	0% (0.91)	33	n/a	n/a
	Hypoxic-CT	9	248		-2.32 (-3.27, -1.37)	< 0.01	0% (0.73)	41	0.072	Low (a,c)
	Hypoxic-HIIT	3	68		-0.88 (-3.40, 1.63)	0.49	0% (0.90)	0	0.904	Very low (a,c,d)
WC	Hypoxic training	11	304		-0.76 (-3.07, 1.54)	0.51	0% (0.99)	50	0.527	Very low (a,c,d)
	Hypoxic-AT	5	140		-0.52 (-3.95, 2.91)	0.77	0% (0.99)	68	0.523	Very low (a,c,d)
	Hypoxic-RT	0								
	Hypoxic-CT	3	72		-0.03 (-4.38, 4.32)	0.99	0% (0.92)	65	0.238	Very low (a,c,d)
	Hypoxic-HIIT	3	92		-0.76 (-3.07,1.54)	0.39	0% (0.50)	55	0.170	Very low (a,c,d)
WHR	Hypoxic training	7	178		-0.02 (-0.04, 0.00)	0.09	0% (0.90)	57	0.775	Very low (a,c,d)
	Hypoxic-AT	2	55		-0.00 (-0.05, 0.04)	0.85	0% (0.82)	62	n/a	n/a
	Hypoxic-RT	0								
	Hypoxic-CT	3	62		-0.01 (-0.06, 0.04)	0.59	0% (0.84)	2	0.961	Very low (a,c,d)
	Hypoxic-HIIT	2	61		-0.03 (-0.06, 0.00)	0.06	0% (0.39)	100	n/a	n/a

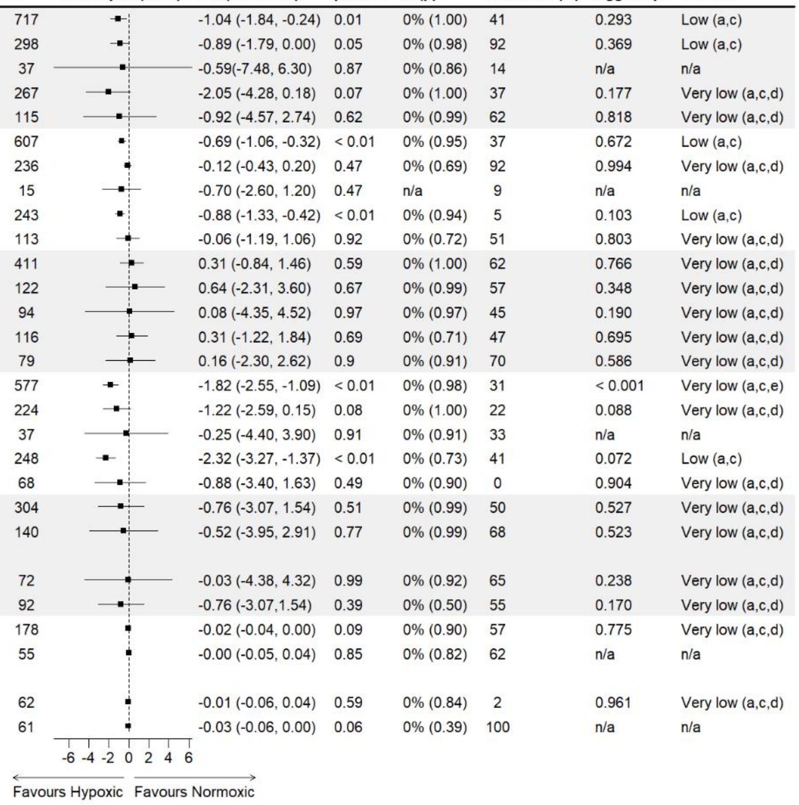

Body composition outcomes in pairwise meta-analyses. *Hypoxic-AT* hypoxic aerobic training; *Hypoxic-RT* hypoxic resistance training; *Hypoxic-CT* hypoxic resistance combined with hypoxic aerobic training; *Hypoxic-HIIT* hypoxic high-intensity interval training. *BM* body mass; *BMI* body mass index; *FM* fat mass; *FFM* fat-free mass; *WC* waist circumference; *WHR* waist-to-hip ratio. *REM* Random effects model, *ROB* risk of bias (percentage of studies with low), *MD* mean difference, GRADE certainty ratings, *very low* the true effect is probably markedly different from the estimated effect, *low* the true effect might be markedly different from the estimated effect, *moderate* the true effect is probably close to the estimated effect, *high* the true effect is similar to the estimated effect. [a]: down rating for risk of bias, [b]: down rating for inconsistency, [c]: down rating for indirectness, [d]: down rating for imprecision, [e]: down rating for publication bias (using Egger's *p*-value).

Supplement 10: Meta regression

Contrast to control	Sample size			Year of publication			Body Mass Index			Proportion of females			Age		
	n	*β*	*p*	n	*β*	*p*	n	*β*	*p*	n	*β*	*p*	n	*β*	*p*
**Body mass**															
Hypoxic-AT	13	-0.07	0.509	13	-0.09	0.458	12	-0.67	0.480	13	-0.01	0.992	13	0.01	0.904
Hypoxic-CT	10	-0.02	0.690	10	-0.02	0.949	10	1.58	0.659	10	-0.72	0.798	10	0.00	0.990
**Body mass index**															
Hypoxic-AT	10	-0.11	0.099	10	-0.00	0.947	10	-0.26	0.743	10	0.47	0.322	10	0.04	0.215
**Fat mass**															
Hypoxic-AT	10	-0.01	0.862	10	0.04	0.922	10	0.08	0.751	10	1.87	0.270	10	0.07	0.220
**Triglycerides**															
Hypoxic-AT	10	-0.09	0.862	10	-0.05	0.958	10	-2.98	0.808	10	19.31	0.439	10	0.31	0.593

Contrast to control	Hypoxia severity			Hypoxia duration			Training weeks			Exercise frequency		
	n	*β*	*p*	n	*β*	*p*	n	*β*	*p*	n	*β*	*p*
**Body mass**												
Hypoxic-AT	13	51.32	0.786	13	-0.01	0.883	13	-0.04	0.821	13	-0.76	0.319
Hypoxic-CT	10	5.16	0.980	10	-0.02	0.532	10	-0.05	0.897	10	-1.97	0.653
**Body mass index**												
Hypoxic-AT	10	-52.14	0.361	10	-8.4	0.251	10	0.02	0.746	10	-0.99	0.093
**Fat mass**												
Hypoxic-AT	10	-47.31	0.669	10	0.03	0.710	10	0.04	0.705	10	-0.74	0.290
**Triglycerides**												
Hypoxic-AT	10	-1562	0.268	10	0.17	0.790	10	1.49	0.238	10	-4.97	0.350

Note We conducted a meta-regression when at least 10 studies contained the same indicators. *Hypoxic-AT* hypoxic aerobic training *Hypoxic-RT* hypoxic resistance training *Hypoxic-CT* hypoxic resistance combined with hypoxic aerobic training *Hypoxic-HIIT* hypoxic high-intensity interval training.
